# Brazil climate highlights 2023

**DOI:** 10.1111/nyas.15394

**Published:** 2025-06-15

**Authors:** Luana Albertani Pampuch, Paola Gimenes Bueno, Michelle Simões Reboita, Ana Carolina Nóbile Tomaziello, Ana Maria Pereira Nunes, Andressa Andrade Cardoso, Caio A. S. Coelho, Camila Bertoletti Carpenedo, Francisco das Chagas Vasconcelos, Helber Barros Gomes, Henri Rossi Pinheiro, Hugo Alves Braga, Iuri Valério Graciano Borges, Maria de Souza Custodio, Maria Leidinice da Silva, Marta Llopart, Rosmeri Porfírio da Rocha, Tércio Ambrizzi, Gyrlene Aparecida Mendes da Silva

**Affiliations:** ^1^ Institute of Science and Technology São Paulo State University (UNESP), São José dos Campos São Paulo Brazil; ^2^ Institute of Astronomy, Geophysics and Atmospheric Sciences Universidade de São Paulo, USP, R. do Matão São Paulo Brazil; ^3^ Natural Resources Institute Universidade Federal de Itajubá, UNIFEI Itajubá Brazil; ^4^ Departamento de Ciências Atmosféricas Universidade de São Paulo, USP, R. do Matão São Paulo Brazil; ^5^ Centro de Previsão de Tempo e Estudos Climáticos (CPTEC), Instituto Nacional de Pesquisas Espaciais (INPE) Rodovia Presidente Dutra Cachoeira Paulista Brazil; ^6^ Center for Studies on Climate Change and Variability (NUVEM) Federal University of Paraná (UFPR), R. dos Funcionários Curitiba Brazil; ^7^ Fundação Cearense de Meteorologia e Recursos Hídricos (FUNCEME) Fortaleza Brazil; ^8^ Institute of Atmospheric Sciences (ICAT) Federal University of Alagoas (UFAL) Maceió Brazil; ^9^ Departamento de Geografía Física, Instituto de Geografía Universidad Nacional Autónoma de México (UNAM) México City México; ^10^ Department of Physics and Meteorology, School of Sciences São Paulo State University (UNESP) Bauru Brazil; ^11^ The Abdus Salam International Centre for Theoretical Physics (ICTP) Trieste Italy; ^12^ São Paulo State University (UNESP) Vargem Limpa Brazil; ^13^ Departamento de Ciências Atmosféricas Universidade de São Paulo, USP São Paulo Brazil; ^14^ Sea Institute Federal University of São Paulo, UNIFESP Santos Brazil

**Keywords:** Brazil, extreme precipitation, heatwaves, intense winds, synoptic‐scale cyclones

## Abstract

In the year 2023, the Earth experienced the highest near‐surface temperature anomalies ever recorded until then. In addition, several extreme weather and climate events occurred around the world, including in Brazil. In this context, the primary objective of this study is to analyze the anomalous temperature and precipitation patterns observed in Brazil during 2023, along with the most significant extreme events. Different datasets and methodologies were applied. The north coast of the state of São Paulo had the highest accumulation of rainfall recorded in Brazil in a single day. September, October, and November 2023 experienced the large precipitation deficits over the Amazon region, leading to a very intense and prolonged drought. The south of Brazil was affected by a large amount of precipitation in a short time, associated with cyclones, resulting in fatalities and economic losses. Southeast and Central‐West Brazil experienced two intense heatwaves in the austral spring, breaking daily temperature records in major cities like São Paulo and Rio de Janeiro. Overall, this study describes the main physical processes responsible for these extremes, along with the socioenvironmental impacts caused by most of them.

## INTRODUCTION

As reported by the Intergovernmental Panel on Climate Change,^1^ the global average temperature has risen by 1.1°C between 2011 and 2020, compared to the 1850–1900 period. This rise is notably more intense over landmasses than over oceans, with various regions around the world experiencing distinct temperature shifts. From the 1970s onward, the global average temperature has escalated at a swifter rate than during any other 50‐year span within the last 2000 years.^1^

According to the World Meteorological Organization,^2^ the globe experienced in 2023 its highest global temperature anomaly ever recorded until then: 1.45°C warmer than pre‐industrial levels (1850–1900) and 0.81°C warmer than the 1991–2020 climatology, surpassing previous records set in 2016 (+1.29°C) and 2020 (+1.27°C). The months from June to December 2023 marked the hottest period on record. September 2023 stood out with the highest global average temperature anomaly (+0.93°C compared to the 1991–2020 climatology), followed by October, November, and December, each setting the second‐highest records (with anomalies of +0.85°C in each month).^3^

Every day of the year 2023 recorded a global average temperature anomaly of 1°C above pre‐industrial levels, with over half of these days experiencing anomalies above 1.5°C and 2 days (November 17 and 18) surpassing 2°C, being the first time this has occurred.^3^ These records were alarming, considering that the Paris Agreement (2016)^4^ aims to limit the increase in global average temperature to a maximum of 1.5–2°C. It is unequivocal that human actions associated with greenhouse gas emissions have contributed to global warming.^5^ In 2023, atmospheric concentrations of carbon dioxide and methane continued to rise and reached record levels (419 ppm and 1.902 ppb, respectively).^3^

According to the National Institute of Meteorology^6^ of Brazil, the temperature was 0.69°C above the 1991–2020 climatology of 24.23°C, making it the hottest year. This record surpassed the highest anomalies ever recorded in the country: in 2015 (+0.67°C), 2019 (+0.61°C), 2016 (+0.43°C), and 1998 (+0.37°C). In 9 months of the year 2023, the average temperature anomaly was above the historical average, with September 2023 recording the highest anomaly (+1.6°C), following the global pattern.

In addition to the record in air temperatures in 2023, the oceans also recorded elevated sea surface temperature (SST). Global SSTs were elevated in March, declined in April and May, and increased again from June, reaching 21.02°C on August 23 and 24, 2023, which was higher than the previous record of 20.95°C observed in March 2016 at the end of a strong El Niño event. For the rest of the year, the global average SST remained exceptionally high, surpassing previous records for warmest years. The Pacific Ocean was characterized by a rapid transition from La Niña to El Niño. The La Niña lasted for 3 years, and by July 2023, the warm phase of the phenomenon was already established.^3^ El Niño conditions became well established in both the ocean and atmosphere only by early September, and a strong El Niño had developed by the end of the year, with the Oceanic Niño Index reaching 2.0°C during the November–January period, marking the highest value recorded since the 2015/2016 El Niño event.^2^

Precipitation in 2023 exhibited distinct characteristics across the globe. Accumulated precipitation exceeded the long‐term average across East and Central Asia, parts of northern Asia, the western Indian summer monsoon region, and portions of the Maritime Continent. Increased precipitation was also observed in northern New Zealand, parts of West, Central, Southern, and East Africa, as well as Western, Central, and Southeast Europe, Southern Scandinavia, and the Western Middle East. Additionally, the above‐average precipitation was recorded in Northwest, Southwest, and Southeast North America, the Greater Antilles, and parts of Southeast South America. Conversely, significant rainfall deficits were recorded in Southeast South America, the Amazon Basin, and much of Central America, as well as in southern Canada, the western Mediterranean region, and Southwest Europe. Other regions affected by below‐average precipitation included parts of Northwest, Central, and Southern Africa, sections of Central Asia, the eastern Indian monsoon region, parts of Southeast Asia and the Maritime Continent, southwestern and coastal northern Australia, and several Pacific Islands.^2^

A large number of extreme events were recorded worldwide in 2023, such as heatwaves over China, North America, Europe,^7^, ^8^ and Brazil;^9^ floods over Africa;^10^ droughts over West Asia^11^ and South America,^12^ especially over the Amazon River Basin;^13^ and wildfires in Canada^14^ and Hawaii.^12^ Heatwaves are among the most severe climate extremes, with increasing frequency, intensity, and duration under global warming, posing major challenges for societies and ecosystems. Despite growing research, significant knowledge gaps remain regarding their underlying physical mechanisms, particularly the interactions between thermodynamic and dynamical drivers across spatial and temporal scales. According to a comprehensive review,^15^ the complexity of heatwave formation arises from the interplay between global factors such as greenhouse gas forcing and regional influences like land‐use changes, aerosols, and soil moisture feedbacks. The authors emphasize the need for coordinated efforts to refine the definition of heatwaves, improve their model representation, feedbacks, and better understand the connections between atmospheric and marine heatwaves. Their work delineates key priorities for future research, including the development of process‐based approaches and interdisciplinary tools to enhance forecasting and impact assessments of heatwaves in a changing climate.

The study by Zhang et al.^12^ summarizes the most significant weather and climate events worldwide in 2023, highlighting that many characteristics of these recent extreme events align with projected future changes in a warming climate. In 2023, they were driven by various phenomena operating at different time scales, including recurring La Niña episodes, monsoons, and cyclones.^12^ Cyclones played a major role in many of these extreme precipitation events. For instance, different types of synoptic‐scale cyclones in the southwestern South Atlantic Ocean^16^, ^17^ are responsible for high waves,^18^ affecting maritime transport, oil platforms, and coastal ecosystems. In addition, abrupt changes in weather conditions, including heavy rainfall and strong winds, can cause economic losses and, in some cases, loss of life in coastal regions, as occurred in 2023.^19^ Besides these phenomena, extreme heat and prolonged droughts also occurred in 2023, with several events accompanied by widespread wildfires. All these events can impact human health, ecosystems, biodiversity, and infrastructure, posing significant risks to water and food availability, public health, and overall well‐being.^2^

Record temperatures and precipitation were also registered in Brazil in 2023. Araçuaí (Minas Gerais state) witnessed, on November 19, 2023, the highest temperature ever recorded in the country of 44.8°C (Instituto Nacional de Meteorologia, or INMET). The previous highest value was in Teresina (Piaui state), on November 21, 2005, reaching a maximum of 44.7°C. Bertioga (São Paulo state) recorded the highest precipitation accumulations ever registered in the country by INMET and the National Center for Monitoring and Alerting of Natural Disasters (CEMADEN): 683 mm in a 24‐h period between February 18 and 19, 2023.^20^ The previous record occurred in 2022 in Petrópolis (Rio de Janeiro state) of 534.4 mm,^21^, ^22^, ^23^ and in 1991 in Florianópolis (Santa Catarina state) of 404.8 mm (INMET). Another highest rainfall accumulation in 24 h was of 346.6 mm that occurred on January 5, March 10, and December 12 and 31, 1999, observed over the mean domain on the central‐north coast of the São Paulo state that includes the Bertioga.^24^ In 2023, two extratropical cyclones were also responsible for at least 54 fatalities in southern Brazil, mainly due to the high amounts of precipitation.^19^, ^25^

Associated with extreme precipitation and temperature in Brazil, the occurrence of natural disasters has been increasing in the country. According to CEMADEN, the country recorded 1341 disaster events in 2023, with 815 of hydrological origin and 526 of geological origin. The number of events is the highest ever recorded since the beginning of CEMADENS's operation in 2011. The region with the highest number of occurrences was the southeast of Brazil. It is estimated that the economic losses associated with these events were nearly US$5 billion, with 74,000 people left homeless, 500,000 displaced, 9263 people injured, and 132 lives lost.^21^

The Climate Study Group (GrEC) from the University of São Paulo (USP)—Brazil also conducts climate monitoring and forecasting for Brazil and monthly highlights the occurrence of extreme events^26^ that can be accessed at http://www.grec.iag.usp.br/. The aim of this study is to discuss the anomalous and extreme temperature and precipitation events observed in Brazil in 2023, as well as to explore atmospheric–oceanic circulation anomalies that contributed to the four selected intense events. The selected events are (1) the unprecedented extreme rainfall (above 650 mm day^−1^) observed on the coast of São Paulo state; (2) the drought in the Amazon region; (3) the extreme precipitation events reaching south Brazil; and (4) the record‐breaking heatwaves in the spring.

## METHODOLOGY

The first two subsections here describe, respectively, the data and methods used to analyze temperature and precipitation extremes in Brazil in 2023, and the third to sixth subsections describe in detail the methodologies employed to explore the circulation–oceanic circulation associated with the extreme selected events.

### Data

Brazil is the study area, and the data used in this work are provided by different sources, as summarized in the Supplementary Material (Figure S1). We used observed data from INMET meteorological stations (https://bdmep.inmet.gov.br/); pluviometers from the Environmental Observation Network of the CEMADEN (http://www2.cemaden.gov.br/pluviometros‐automatico/), of the Ministry of Science, Technology and Innovation (MCTI); and atmospheric variables at different pressure levels from European Centre for Medium‐Range Weather Forecasts (ECMWF) v5 reanalysis (ERA5). ERA5 is a reanalysis available in a global grid of 0.25° x 0.25°.^27^ Volumetric soil water layer 1 (0–7 cm) is from ERA‐Land, a reanalysis dataset that offers a high‐resolution (∼9 km), consistent representation of land variable evolution over several decades, improving upon the resolution of ERA5.^28^ We also used data on precipitation and 10‐m winds from Multi‐Sensor Weather (MSWX), which has a high spatial and temporal resolution (0.1° and 3‐h, respectively) bias‐corrected near‐surface atmospheric variables with global coverage;^29^ and temperature at the top of convective clouds from the Geostationary Operational Environmental Satellite‐16 (GOES‐16) and National Oceanic and Atmospheric Administration (NOAA) SST in a global grid of 0.05° x 0.05°.^30^

### Temperature and precipitation indices

The temperature and precipitation anomalies in Brazil during the year 2023 were investigated using data recorded at conventional meteorological stations from INMET. The variables of daily average temperature, maximum temperature, and daily precipitation were collected from 625 stations, covering the period from 1961 to 2023. Data quality control for missing data was applied only for the period from 1991 to 2023. Series with more than 10% of missing data were excluded. We did not assess earlier years because historical information is scarcer, and we wanted to retain as many stations as possible in the study. The quality control selected 69 stations for compensated average temperature, 93 for maximum temperature, and 118 for precipitation (Figure S2).

Annual and monthly anomalies for temperature and precipitation at each station were calculated for 2023, using the 1991–2023 period as the reference. The records in 2023 of daily mean temperature and maximum temperature were investigated for the available period of each station, since 1961.

Temperature indices defined by the Expert Team on Climate Change Detection and Indices (ETCCDI^31^, ^32^) were used to investigate the extreme events. For temperature, the Warm Spell Duration Index (WSDI) was calculated to identify the heatwaves in 2023. WSDI is calculated first by defining the daily maximum temperature on day *i* in period *j* (*TXij*) and the 90th percentile for the calendar day centered on a 5‐day window (*TXin*90), based on the climatology of 1991–2020. The number of days where *TXij*>*TXin*90 for at least 6 consecutive days is the WSDI. Additional indices were computed to provide other aspects of the heatwave events, based on the WSDI definition of a heatwave, including: the yearly number of heatwaves events (HWN); the length of the longest heatwave event of the year (HWD); and the average magnitude of heatwave (HWM), which is obtained by averaging the maximum temperatures of all heatwave days during 2023.^33^ The ETCCDI indices have been extensively applied to assess past and projected changes in temperature and precipitation extreme events using observational and climate model data at global and regional scales.^34^, ^35^, ^36^

### Wet event on the coast of São Paulo state

For the event that reached the coast of São Paulo, the precipitation data recorded by pluviometers from CEMADEN were used from February 18 to 19, 2023. From CEMADEN stations, we selected only one rain gauge for each city on the coast: Bertioga, São Sebastião, Guarujá, Ilhabela, Ubatuba, and Caraguatatuba that recorded the highest accumulation of rainfall between February 18 and 19. The synoptic conditions were evaluated using variables from the ERA5: mean sea level pressure, geopotential height, zonal and meridional wind, air temperature, relative vorticity, and omega. Daily SST data from NOAA were also utilized.

The tops of convective clouds, at 06 UTC on February 18 and 19, were identified in the infrared channel images (CH13, 10.35 µm) captured by the Advanced Baseline Imager (ABI) sensor on board the GOES‐16. The ABI sensor data set is available at 10‐min intervals, with a spatial resolution of 2 km, and it is processed by the Center for Weather Forecasting and Climate Studies (CPTEC) of the National Institute for Space Research (INPE).

### Drought in the Amazon region

The Amazon drought event was investigated by producing 2023 anomalies of SST and circulation cross sections (from 1000 to 100 hPa) using ERA5. The anomalous vertical velocity (omega), meridionally averaged between 10°N and 10°S, was analyzed for two regions: the western Pacific–northern South America (10°N–10°S, 150°E–40°W) and the Atlantic–northern South America (40°N–25°S, 70°W–40°W). The anomalies for September, October, and November 2023 were computed relative to the 1991–2020 climatology.

### Extreme precipitation in the South of Brazil

A large portion of precipitation in the southern region of Brazil, which comprises the states of Rio Grande do Sul (RS) in the extreme south and Santa Catarina (SC) and Paraná (PR) to the north, was driven by the passage of cyclones.^37^ In 2023, some of these cyclones caused significant impacts on society, including flooding, financial losses, and many fatalities. In this study, cyclones were identified using the algorithm developed previously,^16^, ^38^ referred to as the S2R‐Vortrack code.^39^ The identification process was based on the relative vorticity of the wind at 10 m (negative values in the Southern Hemisphere) and involves three key stages.

First, the cyclone was identified by the minimum relative vorticity using the nearest‐neighbor approach, which must be less than or equal to −1.0 × 10^−5^ s^−1^. The algorithm then corrected the first position by applying a high‐resolution grid within a 250 km radius. This position was used in the second time step as a reference to find the second position. The system's velocity of displacement was then calculated as the difference between two positions, and this velocity was used as the first guess to find the next position by searching for the minimum relative vorticity nearby. This procedure was repeated until a maximum of 10 days was reached and a minimum cyclone lifecycle of 1 day. The tracking algorithm identified all cyclones (all systems with vorticity lower than −1.0 × 10^−5^ s^−1^) in the region, without distinguishing between tropical, subtropical, or extratropical systems. However, since we applied filters, most of the identified systems were synoptic scale, eliminating mesoscale systems. In the southeastern region of Brazil, for example, 20% of the cyclones that occurred in this region during summer were subtropical. Subtropical cyclones mainly developed over the southeastern coast of Brazil, as observed by previous studies.^40^, ^41^, ^42^, ^43^

Cyclones were tracked using wind field data at 10 m height from the ERA5 reanalysis to obtain the 1991–2020 climatology and the 2023 cyclogenesis events, marking the first identification of each cyclone. Cyclogenesis density is defined as the number of cyclogenesis events occurring within a 3° x 3° latitude by longitude grid. This density is calculated by dividing the total number of events by the grid area in square kilometers, multiplied by 10⁵, and then dividing by the number of years to determine the annual average.^39^, ^42^, ^44^ We discussed the annual mean of cyclogenesis for 2023, along with the anomaly, which is represented by the difference between 2023 and the 1991–2020 climatology.

Additionally, we selected the four most intense cyclones of 2023 to analyze their associated accumulated precipitation and their relative contribution to monthly precipitation, maximum wind speed during their lifecycle, and the maximum difference between daily precipitation during cyclone days and the climatological 95th percentile (P_95_). These analyses were conducted using MSWX data,^29^ and the cyclones occurred during June 14–22, July 12–16, September 2–5, and October 3–6.

The accumulated precipitation during each cyclone's lifecycle was calculated by summing the daily precipitation values. The relative contribution—the accumulated precipitation for the days within the cyclone's period—was compared to the total monthly precipitation for the same month. The result was expressed as a percentage, indicating how much of the total monthly precipitation was associated with each cyclone event. The maximum wind speed throughout the lifecycle was identified by determining the highest wind speed recorded each day of the cyclone's life. For the analysis, we focused on the wind field on the day when the highest wind speed was observed over the continent during the cyclone's lifecycle. The P_95_ value was derived from daily precipitation data from 1991 to 2020 over the southern Brazil domain (35°S–22.1°S, 59°W–46.1°W). The difference between the daily accumulated precipitation on cyclone days and the P_95_ threshold was then calculated, reporting the largest observed value over the continent.

The impacts of the cyclones were obtained by utilizing the damage management report from the Integrated System of Information on Disasters^45^ of the Brazilian Civil Defense, administered by the Ministry of Integration and Regional Development of Brazil, with a specific focus on extreme climatic events occurring in the southern region of the country. Furthermore, the damage caused by convective storms, flash floods, floods, and landslides was selected. The volumetric soil water layer 1 (0–7 cm) of the ECMWF Integrated Forecasting System was also used on an hourly time scale (00, 06, 12, 18), and daily averages were calculated. This analysis was conducted to investigate soil behavior during a cyclone event that did not have a large rainfall accumulation but still caused a significant number of disasters.

### The record‐breaking heatwaves of Spring 2023

Variables from ERA5 reanalysis were used to investigate the large‐scale circulations associated with heatwave episodes. Air temperature at 2 m (t2m) was used to verify whether the ERA5 reanalysis reproduces the spatial patterns and magnitude associated with heatwaves. The 500‐hPa geopotential height, 200‐hPa zonal wind speed, and specific humidity were used to characterize circulation anomalies associated with heatwaves. Daily data for atmospheric variables were used to calculate the anomalies based on the seasonal (September–October–November) 1991–2020 climatology. These anomalies incorporated the two prolonged heatwave events observed on September 17–27 and November 11–18, 2023. The choice of the periods was based on the analysis of heatwave events identified at the Mirante de Santana meteorological station in São Paulo, Brazil (not shown), considering periods with maximum temperatures above the 90th percentile for a calendar day centered on a 5‐day window for at least 6 consecutive days. Additionally, we calculated daily average anomalies of maximum surface air temperatures for Central–West and Southeast Brazil during the austral spring of 2023 (September 1–November 30). These anomalies were derived from the data collected at 36 INMET meteorological stations within these regions.

## RESULTS

### Brazil in 2023

**FIGURE 1:**
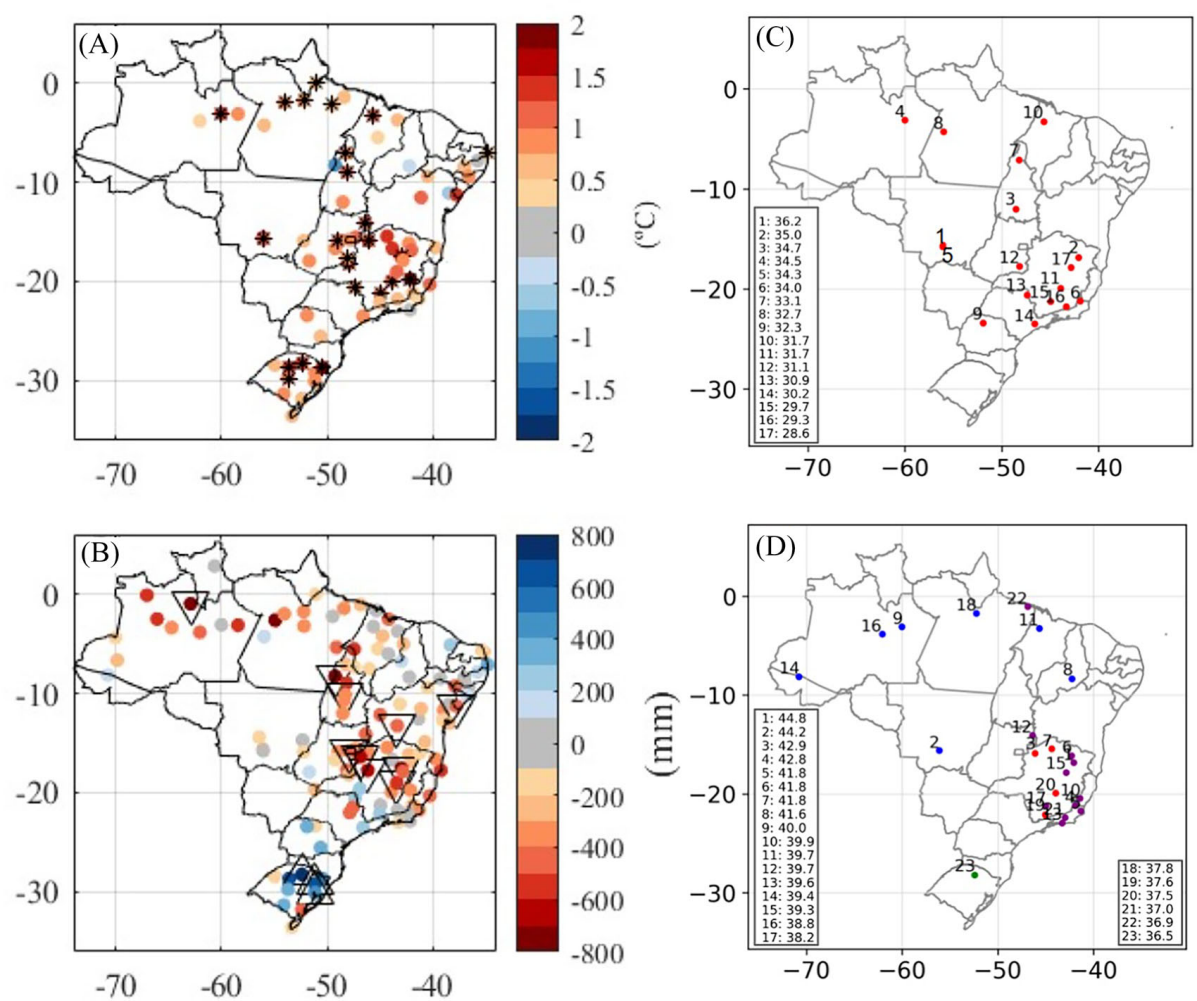
Records of Instituto Nacional de Meteorologia (INMET) conventional meteorological stations: (A) 2023 annual anomalies of compensated average temperature (°C) (the stations where the highest temperature anomaly was recorded in 2023 are marked with (*); (B) 2023 annual anomalies of precipitation (mm) (stations with highest precipitation anomaly recorded in 2023 are marked with upward triangles, while the lowest precipitation anomaly recorded in 2023 are marked with downward triangles); (C) 2023 records of air temperature (°C); and (D) 2023 records of maximum temperature (°C), with four events in September (red), seven in October (blue), 11 in November (purple), and one in December (green).

The annual anomaly of compensated average temperature in 2023 was positive for almost all the stations used (92.75%), with the anomaly recorded as the highest in 25 of them since 1961 (stations marked with *), indicating a warming pattern in Brazil in 2023 (Figure 1A). Comparing the average of the annual anomalies for all 69 stations, 2023 stands out as the hottest year (+0.84°C), followed by 2019 (+0.76°C) and 2015 (+0.74°C). The annual precipitation anomaly was positive in the south and at some stations in the Southeast and Northeast of Brazil. In other parts of the country, negative anomalies were recorded (Figure 1B). In 2023, three stations in Rio Grande do Sul state (Southern Brazil) experienced the highest precipitation anomalies (marked with upward triangles), while 10 stations in the North, Northeast, and Southeast of Brazil recorded the lowest precipitation anomalies (marked with downward triangles).

Figure 1C,D presents the stations with the highest temperatures in the series. In 2023, 17 (24.64%) out of 69 stations set a record for compensated average temperature (Figure 1C), and 23 (24.73%) out of 93 recorded a record for maximum temperature (Figure 1D). The records for maximum temperature occurred four times in September, seven in October, 11 in November, and one in December. The highest maximum temperature of 44.8°C ever recorded in Brazil was observed in Araçuaí (MG) on November 19 (Figure 1D).

**FIGURE 2:**
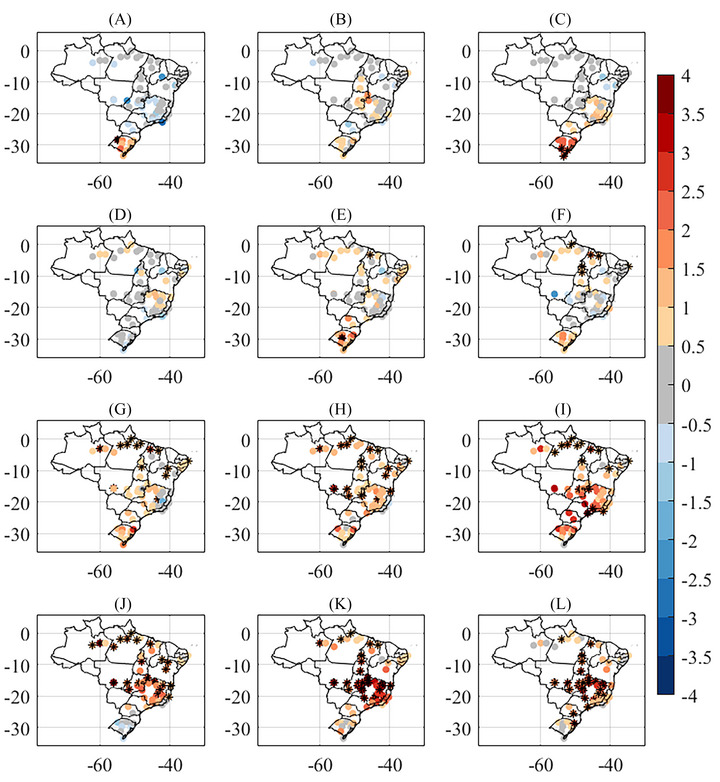
Compensated average temperature (°C) monthly anomalies for 2023 for Instituto Nacional de Meteorologia (INMET) conventional meteorological stations using 1991–2020 climatology, for (A) January, (B) February, (C) March, (D) April, (E) May, (F) June, (G) July, (H) August, (I) September, (J) October, (K) November, and (L) December (in °C) (the stations with the highest temperature monthly anomaly are marked with *).

For the monthly anomalies of compensated average temperature, it is possible to observe that, in January (Figure 2A), only RS state registered warmer conditions, while the rest of the country had temperatures within or below average. In February and March (Figure 2B,C), the warmer temperatures extended to the southeast and central‐west regions of the country. In April (Figure 2D), RS state had below‐average temperatures, while parts of the northeast, north, and southeast showed positive anomalies. In May and June (Figure 2E,F), positive anomalies intensified in southern Brazil, with monthly records in both the south and north (marked with *). From July onward (Figure 2G–L), the country experienced higher positive temperature anomalies, with several monthly records (marked with *) and the largest monthly anomalies observed in 2023 (except for October, which had negative anomalies in RS).

**FIGURE 3:**
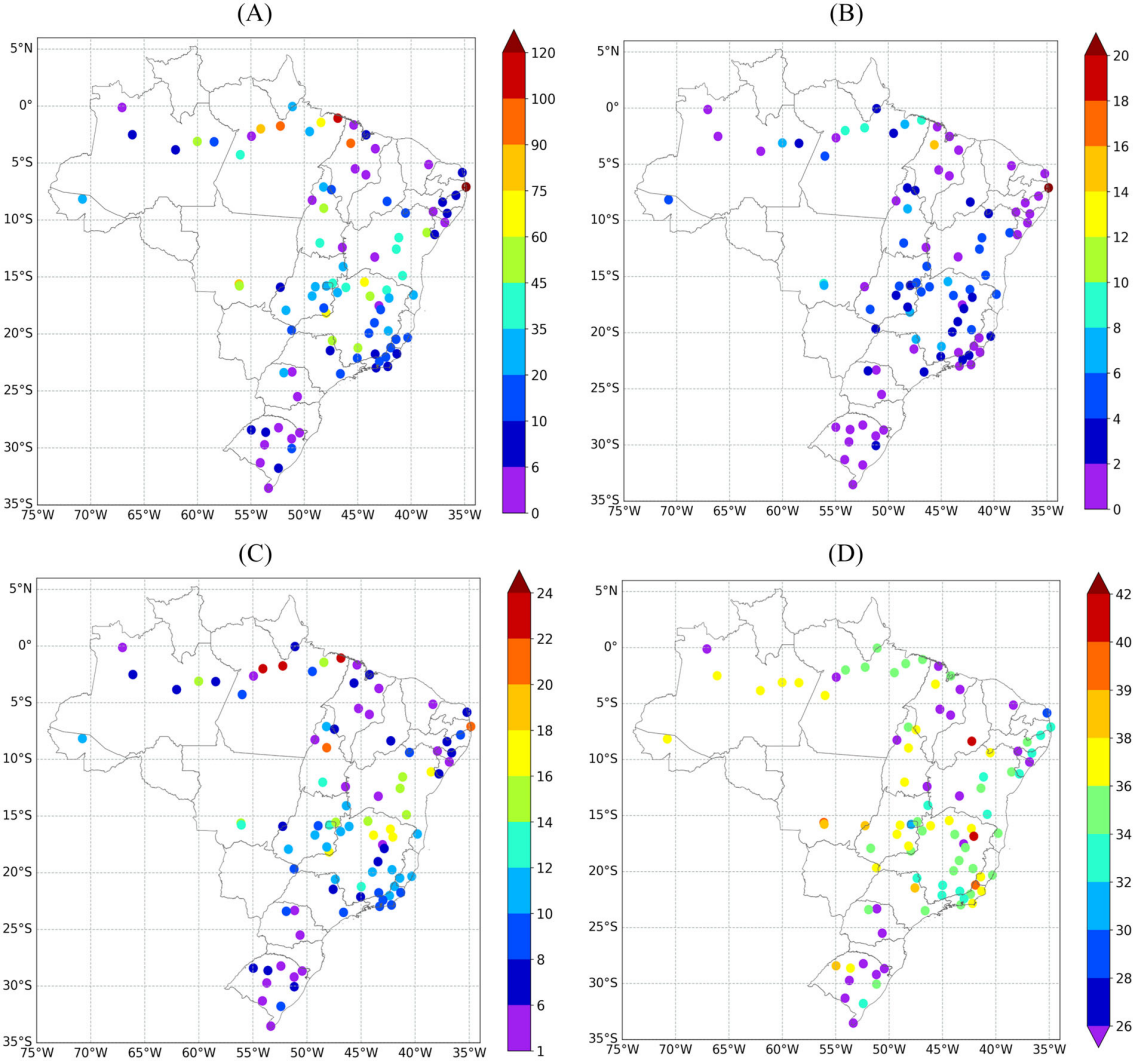
(A) Warm Spell Duration Index (WSDI, in days); (B) yearly number of heatwaves (HWN); (C) length (in days) of the longest heatwave of the year (HWD); and (D) average daily magnitude (in °C) of all heatwaves events (HWM) during 2023.

Regarding heatwave events, the highest values of WSDI were recorded at INMET stations located in the central–northern region of Brazil. In 2023, one station in the state of Pará and another in the state of Paraíba recorded 8 and 20 heatwave events, respectively, which together total more than 100 days (WSDI) at each station (Figure 3A). The highest occurrences of heatwaves during the year (HWN) were observed in stations in the states of Pará, Maranhão, and Paraíba (Figure 3B). The station in Paraíba recorded the highest number of occurrences, with 20 heatwave events, and also registered the highest WSDI, totaling 160 heatwave days. Most of the stations located in the states of Bahia, Tocantins, Goiás, and Minas Gerais recorded between 2 and 8 heatwave events throughout the year. The most prolonged heatwaves (HWD) also occurred at stations in the central‐northern region of Brazil (Figure 3C). Some stations in the north and northeast regions recorded events lasting over 20 days, with two stations in the state of Pará recording heatwave episodes lasting 23 days. The two most intense heatwaves recorded a maximum average temperature of 40°C (Figure 3D), one in the state of Piauí and the other in the state of Minas Gerais. This latter was associated with the highest maximum temperature ever recorded in Brazil (Figure 3B), which is also analyzed in the section “The record‐breaking heatwaves of Spring 2023”.

**FIGURE 4:**
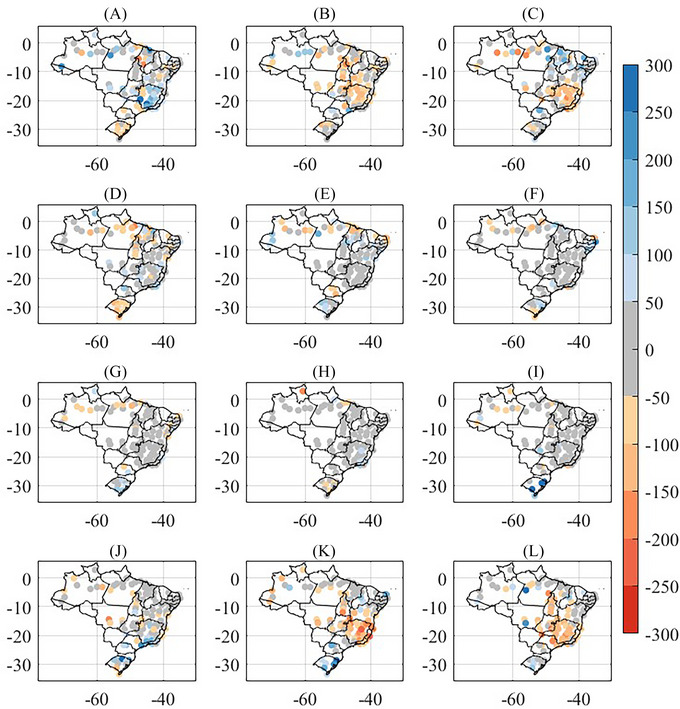
Precipitation monthly anomalies for 2023 for Instituto Nacional de Meteorologia (INMET) conventional meteorological stations using 1991–2020 climatology: (A) January, (B) February, (C) March, (D) April, (E) May, (F) June, (G) July, (H) August, (I) September, (J) October, (K) November, and (L) December (in mm).

In southern Brazil, despite the annual anomaly being positive (Figure 1B), the first 4 months of the year were marked by negative precipitation anomalies (Figure 4A–D). From May onward, positive anomalies began to be recorded (Figure 4E) and intensified between September and November (Figure 4I–K). In southeastern Brazil, the year started with positive precipitation anomalies (Figure 4A), while negative anomalies were observed in February, March, November, and December, with the precipitation deficit reaching up to 250 mm in the last 2 months of the year (Figure 4A–L). In the far north of Brazil and some stations in the Northeast, positive anomalies predominated in January. However, for the rest of the year, negative anomalies prevailed in the north, while conditions remained closer to normal in the northeast (Figure 4A–L).

### Wet event at the coastline of São Paulo state

**FIGURE 5:**
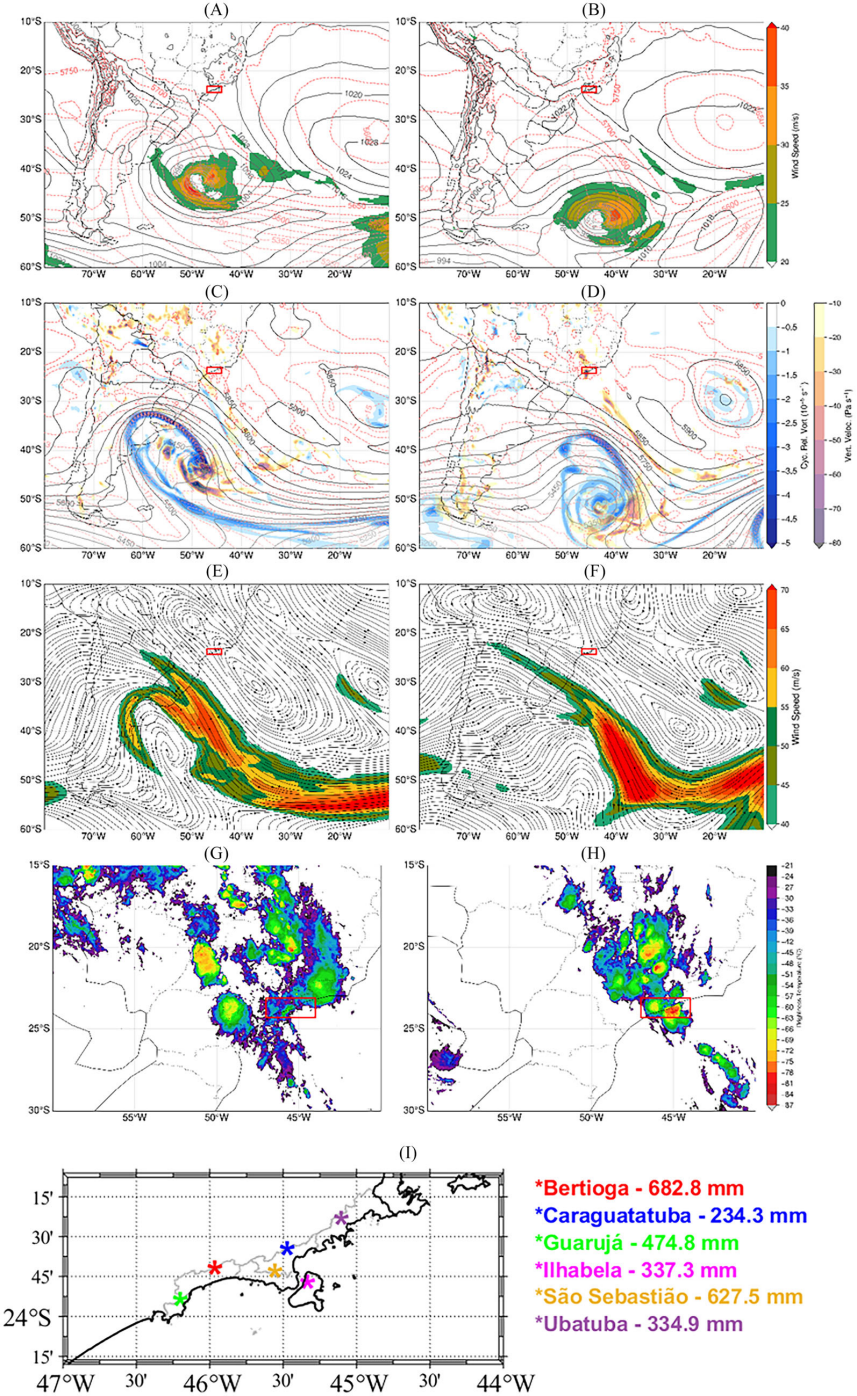
(A, B) Thickness between 1000 and 500 hPa layer (m, red lines), mean sea level pressure (hPa, black lines), and 850‐hPa isotachs (m/s, shaded); (C, D) air temperature (°C, red lines), geopotential height (m, black lines), cyclonic relative vorticity (10^−5^ s^−1^), and omega (hPa s^−1^) at 500‐hPa; (E, F) 250‐hPa streamlines and isotachs (m/s, shaded); (G, H) infrared images (CH13, 10.35 µm) from the GOES‐16 satellite at 00 UTC on February 18 (A,C,E, and G) and 19 (B,D,F, and H). The red box represents the coast of the São Paulo state, highlighting the recorded precipitation totals in the analyzed municipalities shown in (I).

From 15 UTC February 18 to 15 UTC February 19, 2023, on the north coast of the state of São Paulo (red box in Figure 5), the highest accumulation of rainfall recorded in Brazil occurred in a single day: 682.8 mm in just 24 h in the municipality of Bertioga. Other significant accumulations were observed in São Sebastião (627.5 mm), Guarujá (474.8 mm), Ilhabela (337.3 mm), Ubatuba (334.9 mm), and Caraguatatuba (234.3 mm) (Figure 5I). This event caused flooding, coastal erosion, and landslides, resulting in human, environmental, and economic impacts. According to the Civil Defense of the State of São Paulo, 1126 people lost their houses, 1090 people were displaced from their homes, and 65 deaths were registered, as well as several blocked roads, collapsed barriers, and falling trees. Although the highest accumulations of rainfall occurred in Bertioga, the highest number of deaths (64) was observed in São Sebastião due to the great number of people living in precarious settlements and in high‐risk areas, subject to mass movements on the slopes of the mountains of the Serra do Mar.^46^ The study by Marengo et al.^20^ also described this event, but mainly focused on natural disasters.

During this heavy rainfall event, the weather was marked by the passage of a polar cold front over the warmer‐than‐normal subtropical South Atlantic (Figure S3). On February 17–18, this cold front remained stationary over the state of RS, with the associated extratropical cyclone over the South Atlantic Ocean, centered at approximately 52°W–46°S and with a central pressure of 974 hPa (Figure 5A). At upper levels, the jet stream (Figure 5A), the presence of a cyclonic circulation over the coast of the Northeast Region of Brazil, and an anticyclonic circulation over the Central–West Region (Figure 5E) contributed to the pattern and movement of the frontal system. This pattern, which resembles a typical summer circulation over Brazil, was completed by the low‐level at 850 hPa flow from the Amazon toward the coast of São Paulo, which has characteristics similar to the South American low‐level jet, present between February 14 and 17 (Figure S4). This low‐level circulation contributed to increasing the availability of water vapor in the region, providing fuel for the heavy rainfall event. The anticyclonic circulation at upper levels progressively shifted westward on February 18, 12 UTC, while the cyclonic circulation moved toward the continent, where it remained until the end of February 19 (Figure S5). Accompanying the pattern at upper levels, from February 18 onward, the circulation at 850 hPa became disorganized (Figure S4).

On February 19, the frontal system moved eastward, and the extratropical cyclone intensified (Figure 5B,D). The presence of the frontal system favored more intense winds from the adjacent South Atlantic toward the coast of São Paulo (Figure S2). This circulation, combined with the orographic effect of the Serra do Mar, intensified the upward movements (Figure 5D) and contributed to the formation of deep convective clouds (Figure 5F). The South Atlantic, adjacent to the coast of São Paulo, was 1–2°C warmer than average (Figure S3). This resulted in increased heat and moisture flux rates from the sea to the atmosphere, strengthened by the intense winds associated with the cold front.^20^ The combination of the synoptic cold front with local features (mountains of the Serra do Mar and intense near‐surface heat fluxes) resulted in intense and persistent precipitation over the north coast of the state of São Paulo.

### Drought in the Amazon region

**FIGURE 6:**
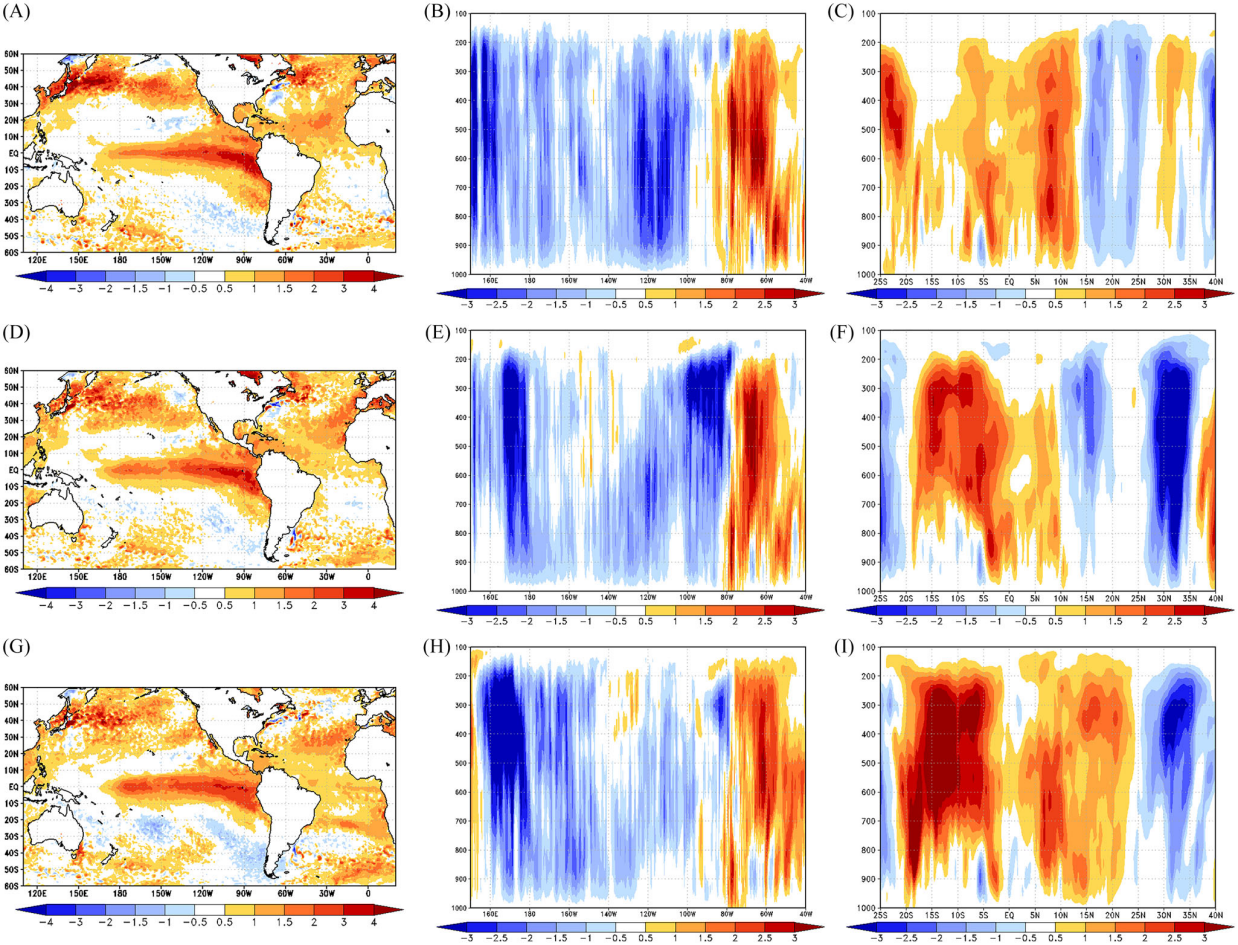
Sea surface temperature anomalies (panels A, D, and G), vertical cross sections of anomalous vertical velocity (omega, 10^−2 ^Pa.s^−1^) meridionally averaged between 10°N and 10°S (panels B, E, and H), and vertical cross sections of anomalous vertical velocity (omega) zonally averaged between 70°W and 40°W (panels C, F, and I) for September (the first row), October (the second row), and November (the third row) 2023. Anomalies were computed with respect to the 1991−2020 climatology using ERA5.

Droughts in the Amazon region have been reported to occur during the combined manifestation of the El Niño phenomenon in the equatorial Pacific Ocean and abnormally warm conditions in the tropical North Atlantic Ocean.^47^, ^48^ During the spring of 2023, a major drought event was observed in the Amazon region, with the Negro River in Manaus recording in November its lowest level of 12.7 m in 121 years. As shown in Figure 4, September, October, and November 2023 experienced large precipitation deficits over the Amazon region, which contributed to such a low Negro River level in Manaus. Figure 6A,D, and G shows that, from September to November 2023, the prevailing large‐scale forcing associated with the observed precipitation deficit in the Amazon were the (a) El Niño phenomenon, with SST anomalies larger than 2°C in parts of the equatorial Pacific Ocean, and (b) anomalous SST warming in the tropical equatorial Atlantic Ocean, with anomalies larger than 1°C in parts of Tropical North Atlantic basin.

Figure 6B,C,E,F,H and I shows vertical cross sections of anomalous vertical velocity (omega) meridionally averaged between 10°N and 10°S (panels B,E, and H), and vertical cross sections of anomalous vertical velocity (omega) zonally averaged between 70°W and 40°W (panels C,F, and I) for September, October, and November 2023. Figure 6B,E and H shows that prevailing subsidence was noticed over the Amazon, as illustrated by positive vertical velocity (omega) anomalies east of 70°W, and prevailing ascending vertical motion was noticed over the warmer‐than‐normal equatorial Pacific, as illustrated by negative vertical velocity (omega) anomalies east of 100°W, indicating an anomalous Walker circulation that is consistent with the observed El Niño conditions. Figure 6C,F and I shows that prevailing subsidence was noticed over the Amazon, as illustrated by positive vertical velocity (omega) anomalies to the south of the equator. Also, prevailing ascending vertical motion was noticed over the north Atlantic, as illustrated by negative vertical velocity (omega) to the north of the equator, depicting an anomalous Hadley circulation consistent with the observed anomalous SST warming in the tropical north Atlantic Ocean.

### Extreme daily rainfall in Southern Brazil

The year 2023 was characterized by high precipitation amounts, especially in the states of RS and SC (Figure 4). Some episodes of daily extreme precipitation were associated with synoptic‐scale cyclones, and a total of four events (which occurred in June, July, September, and October) were selected based on their observed precipitation characteristics and impacts. For the four selected cyclones of 2023, Figure 7 depicts three pieces of information: the accumulated precipitation (panels A‐D), the ratio between this precipitation (panels E‐H) and the monthly accumulated precipitation, and the wind speed for the day with maximum winds over the continent during the cyclone's lifecycle (panels I‐L).

**FIGURE 7:**
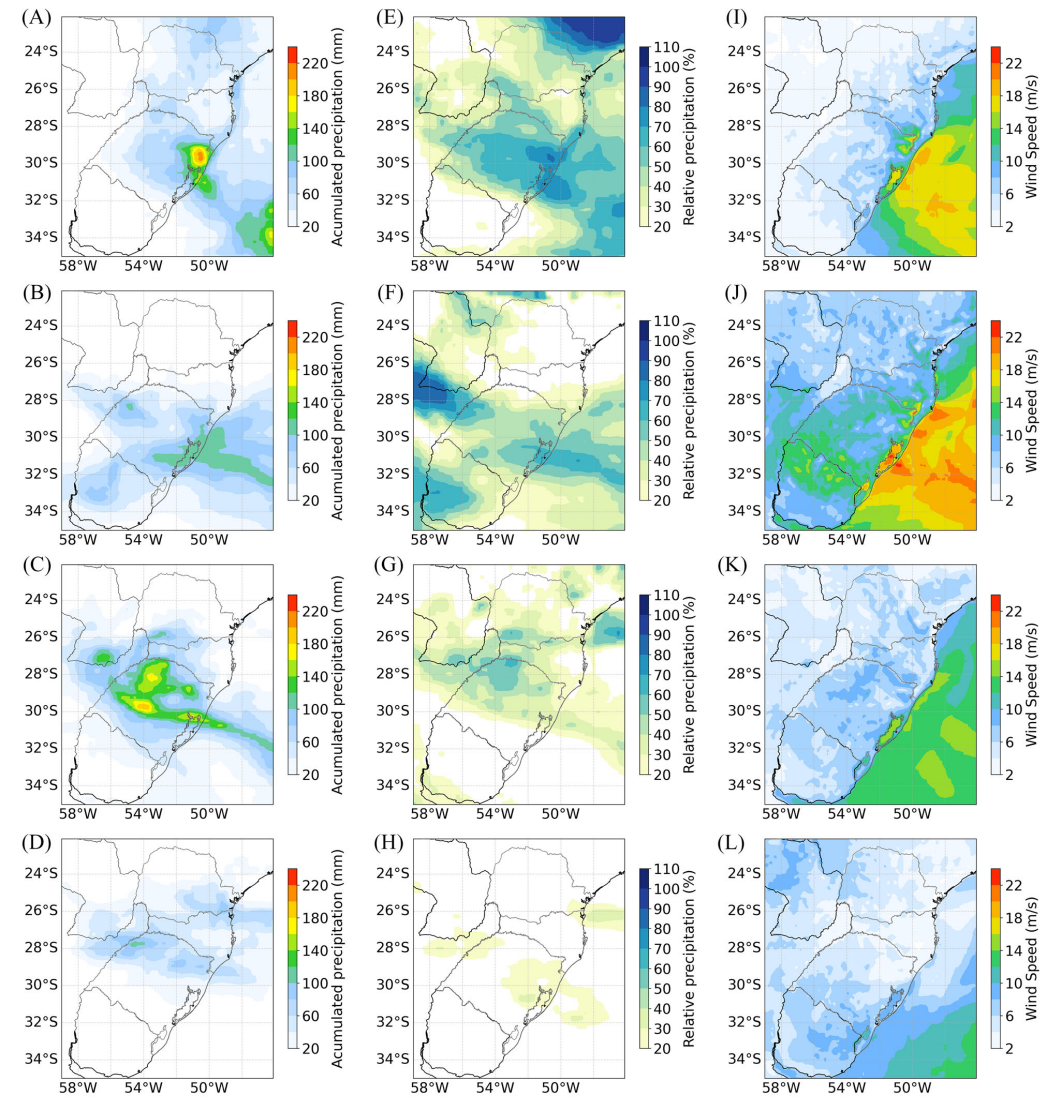
Accumulated precipitation (mm; panels A‐D) from Multi‐Sensor Weather (MSWX) reanalysis, percentage of monthly precipitation (%; panels E‐H), and maximum daily wind speed (m/s; panels I‐L) associated with four selected cyclones of 2023: (A)‐(E)‐(I) June 14–22, (B)‐(F)‐(J) July 12–16, (C)‐(G)‐(K) September 02–05, and (D)‐(H)‐(L) October 03–06.

**FIGURE 8:**
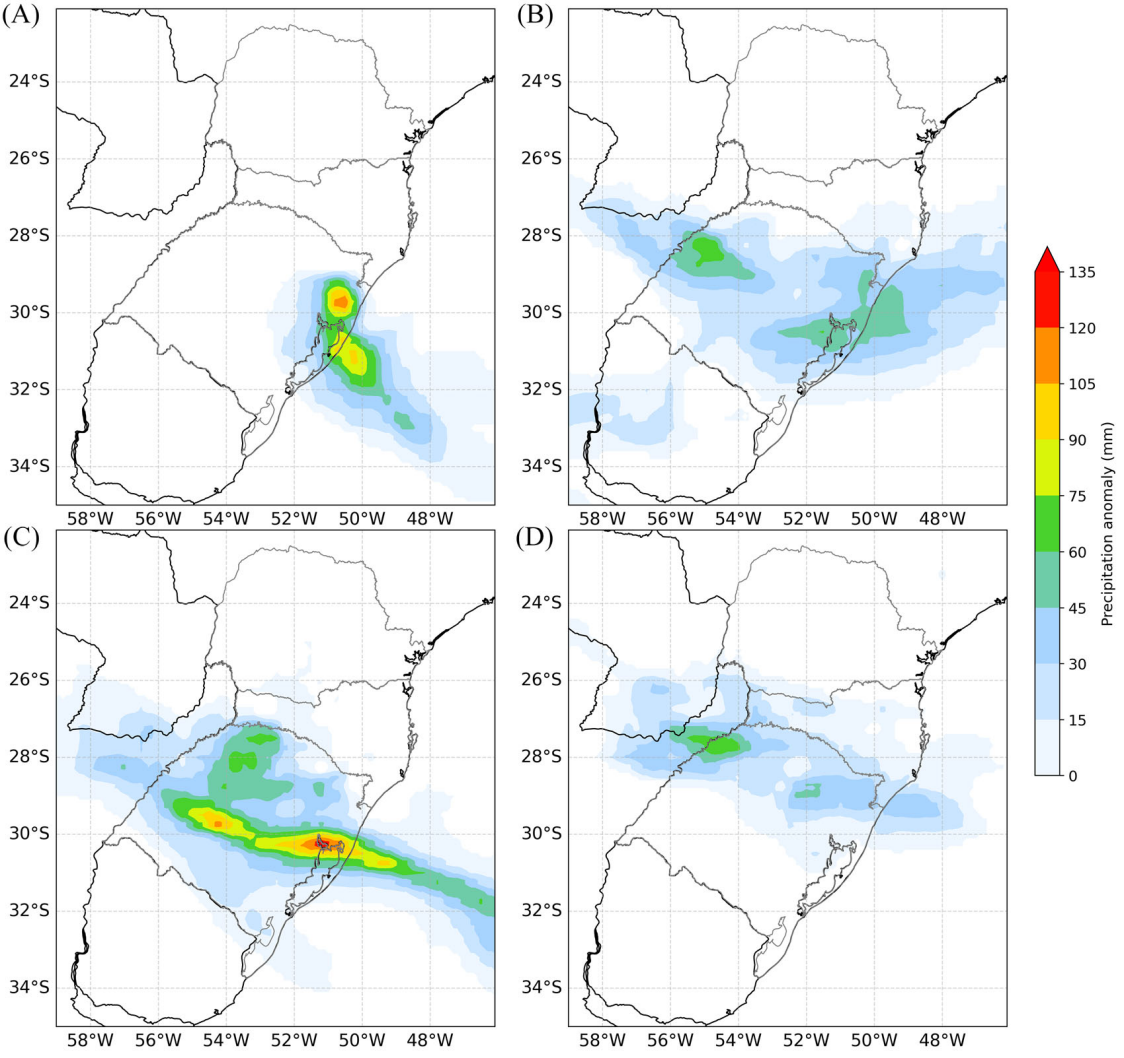
Day with maximum positive difference between daily precipitation and the climatological 95th percentile of daily precipitation (1991–2020) associated with the four cyclones of 2023: (A) June 16, 2023, (B) July 12, 2023, (C) September 4, 2023, and (D) October 4, 2023.

The cyclone from June 14 to 22, 2023, produced a very localized center of intense precipitation (∼240 mm) over the eastern RS (Figure 7A). These 8 cyclone‐days were responsible for most of the monthly precipitation, with above 80% of the monthly mean in the eastern part of RS (Figure 7E). The day with the maximum winds (June 16, Figure 7I) occurred at the same location as the more intense precipitation center, along the RS coast and Laguna dos Patos regions (∼30°S–51° W). The winds reached maxima of around 18 m/s (∼64 km/h), similar to those observed at the Tramandai meteorological station, as shown in Bartolomei et al.^19^ This cyclone was responsible for most of the positive precipitation anomaly shown for the same area previously in Figure 1B. In terms of extremes, for 6 out of the 8 days of the cyclone lifecycle, the daily precipitation exceeded the P_95_ (considering the climatology of the period 1991–2020). Figure 8 presents the difference between the daily precipitation on cyclone days and the P_95_ for the day where this difference was maximum over the continent. The difference exceeded 80 mm/day in a very concentrated core area in the east of RS, which is the most populated area of the region (Figure 8A). The damages associated with this cyclone included at least 16 deaths^19^ and significant economic losses, with more than 10,000 people displaced from their residences.^45^

Although with less accumulated precipitation intensity, July hosted another cyclone impacting South Brazil. It occurred between July 12 and 16, resulting in at least one death^19^ and 1000 people displaced from their residences.^45^ During this event, rainfall accumulation reached approximately 120 mm (Figure 7B), representing ∼60% of the monthly accumulated for most of RS (Figure 7F). Maximum wind speeds associated with this cyclone reached approximately 24 m/s (86 km/h, July 13) in the area covering Laguna dos Patos, the northwest, and the coast of RS (Figure 7J). These wind speeds are similar in different datasets, for instance, MSWX analysis and meteorological station observation at Porto Alegre on July 13 at 12 UTC, which recorded 23 m/s.^19^ Despite the accumulated precipitation amount not being as high as for the June cyclone, the precipitation exceeded the P_95_ for 3 days of the July cyclone. For the day of maximum precipitation, Figure 8B indicates that, in most parts of RS, precipitation exceeded the P_95_ by more than 20 mm/day.

From the 2nd to the 5th of September, another cyclone had a great impact on the south of Brazil, with associated damages including 54 fatalities confirmed by the Civil Defense of Rio Grande do Sul,^25^ and more than 5000 people displaced from their residences.^44^ This event was considered the worst natural disaster in Rio Grande do Sul at the time. The northwest/southeast pattern of rainfall (29°S–33°S and 58°W– 48°W) covering most parts of RS indicates precipitation associated with a cold front (Figure 7C). For the cyclone lifecycle, the accumulation of rainfall over 200 mm was observed in the central areas of RS (Figure 7C), which over the center–north of RS represents up to 60% of its monthly climatology (Figure 7G). Precipitation exceeding the P_95_ was recorded on 3 out of the 4 days of the event, with the largest difference between daily precipitation and the P_95_ occurring in the central part of RS (Figure 8C), where the daily precipitation exceeded the P_95_ by up to 135 mm/day. For the day with maximum winds, velocities of ∼10 m/s (∼36 km/h, September 5) were observed in the region of Laguna dos Patos and the coast of RS (Figure 7K).

In October, a cyclone occurred between October 3 and 6. The accumulated precipitation for this period (Figure 7D) shows values around 100 mm in almost the same region as the September pattern, concentrating more in the north of RS and east of PR, but with much less intensity, since September, as an example, showed values around 200 mm for the same area. This also reflects the percentage of monthly precipitation (Figure 7H), which shows that around 30−40% of that month's precipitation occurred in this period. The maximum wind speed over the continent occurred on October 3 (Figure 7J) in the southern region of Rio Grande do Sul, reaching approximately 10 m/s (36 km/h). Additionally, on October 4, daily precipitation exceeded the 95th climatological percentile by up to 75 mm/day (Figure 8D).

**FIGURE 9:**
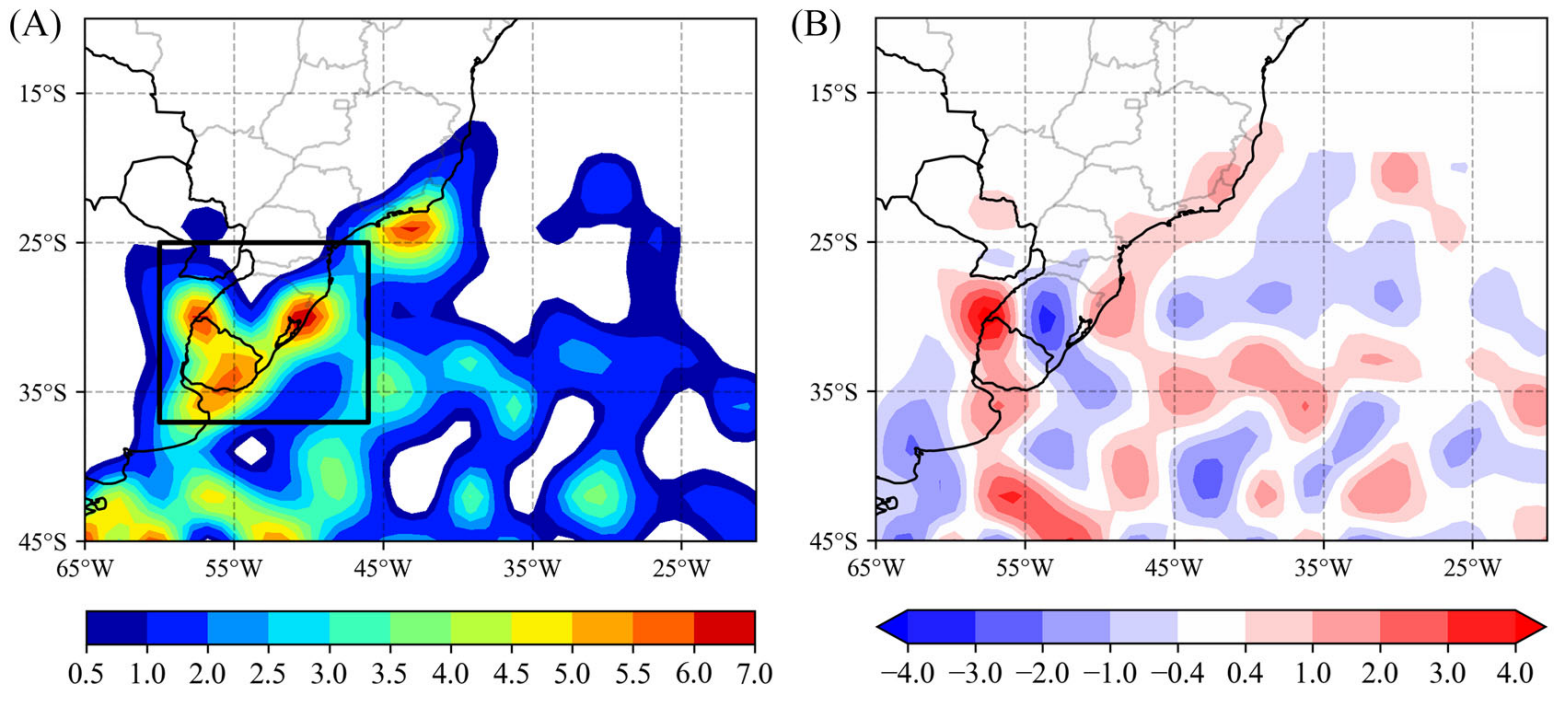
Annual density of genesis: (A) 2023; (B) annual anomaly calculated for 2023 by subtracting the reference period of 1991–2020. The density unit is the number of cyclones per area (km^2^) × 10^5^ per year.

The annual mean and anomaly of cyclogenesis density for 2023 are shown in Figure 9A. The density indicates four main cyclogenesis cores: the South Brazil coast, the Uruguay coast, the region of tripartite frontiers (northwestern Uruguay–southern Brazil–northeastern Argentina), and Southeast Brazil (Figure 9A). These cyclogenetic cores include the cyclogenesis events that moved to the east and affected southern Brazil with heavy precipitation. Compared with climatology, there was, in 2023, less cyclogenesis over the central part of RS, while cyclogenesis increased in the northeast and western sectors of RS (Figure 9B). The high positive anomaly of cyclogenesis in the extreme west and northeast of RS (Figure 9B) may have caused more precipitation in the east, contributing to the increased occurrence of associated cold fronts. The signal of frontal cyclones in the rainfall is indicated by the pattern of the accumulated precipitation that extends in the east–west direction over South Brazil (see Figure 7A‐D).

The predominance of the frontal precipitation pattern can also be explained from a seasonal perspective. Figure S6 shows the seasons of winter (JJA) and spring (SON), which include the cyclones of June and September described previously, displaying a positive anomaly of density to the east and along the coast of South Brazil. This may also indicate anomalies of precipitation concentrated in the eastern part of that region, as well as the elongated east–west pattern for precipitation observed in the four cyclones highlighted so far.

When analyzing the 2023 occurrences of damage associated with extreme weather events across the southern region of Brazil, which include damage caused by convective storms, flash floods, floods, and landslides in each municipality of the states, October recorded the highest number of disasters in the central area,^45^ corresponding to the state of SC. In contrast, the western and northern portions, encompassing the states of RS and PR, experienced the most occurrences between August and November, with the latter registering the highest number of events in both states. Notably, although previous Octobers showed significantly anomalous precipitation, these anomalies did not result in a corresponding increase in disaster events.

This raises a question: If the precipitation associated with the cyclone of October 3–6 did not record an accumulation as substantial as other cyclones, what explains the overwhelming number of disasters in this month? Why October and not the other months, where cyclone events caused the highest precipitation amounts? It may be reasonable to assume that October's cyclone did not play a major part in causing the disasters, or at least it was not the sole factor. From this assumption, it can be hypothesized that the prolonged dry period between March and August, followed by a significant increase in precipitation from September onward, led to a rapid recovery of soil moisture (Figure S7). As a result, by October, the soil may have reached saturation, reducing its ability to absorb additional rainfall and increasing runoff potential. Additionally, the occurrence of another cyclone, which was not as strong as those that occurred before, as for September's, for example, may have been sufficient to trigger these disasters under already vulnerable conditions.

### The record‐breaking heatwaves of Spring 2023

The record‐breaking heatwaves of Spring 2023 marked an unprecedented phenomenon across various regions of Brazil, as previously shown in Figure 1 and Figure 3, consistent with a global trend toward extreme weather events. During this period, two significant heat spells hit a large region of Brazil and neighboring countries, increasing the maximum temperatures by up to 10°C above the climatology in southeastern and central–western Brazil (Figure S8).

As detailed in the Introduction section, the early spring heatwave started in mid‐September and persisted for about 10 days, contributing to the highest temperature anomaly observed in any single month in Brazil. For southeastern and central–western Brazil, Figure S8 shows the time series of daily anomalies in relation to the climatology of 1991–2020 in the maximum air temperatures computed using various meteorological stations. Notably, warmer temperatures persisted throughout the analyzed period for both the central–west and southeast regions of Brazil. The highest temperatures occurred in the second half of September 2023, with positive anomalies exceeding 4°C for 6 consecutive days in both regions.

A subsequent heatwave episode occurred in November, characterized by exceptionally high temperatures exceeding 40°C in several locations. During this event, positive temperature anomalies exceeded 6°C for 9 consecutive days in both regions (Figure S8). As reported by the INMET, many major cities established new record‐breaking temperatures, including Cuiabá (44.2°C), Belo Horizonte (37.5°C), Manaus (40.0°C), and Rio de Janeiro (39.6°C). Araçuaí, in the state of Minas Gerais, recorded the highest temperature ever observed in Brazil (44.8°C, INMET). São Paulo (Mirante do Santana meteorological station) experienced its second‐highest temperature ever recorded on November 13 and 14 (37.7°C), only 0.1°C lower than its historical record set on October 17, 2014 (INMET). The two extraordinary heatwave events ended similarly, that is, by a sudden temperature decline associated with cold front incursions (not shown).

Comparing 2023 with previous years, the period from September to December 2023 in the Mirante de Santana meteorological station in São Paulo (not shown) presented the highest average maximum temperature of 29.5°C, with 48 (16) days exceeding the 90th (99th) percentile. These results are in agreement with previous studies^49^, ^50^ that also observed positive trends in heatwave frequency since the 1980s.

**FIGURE 10:**
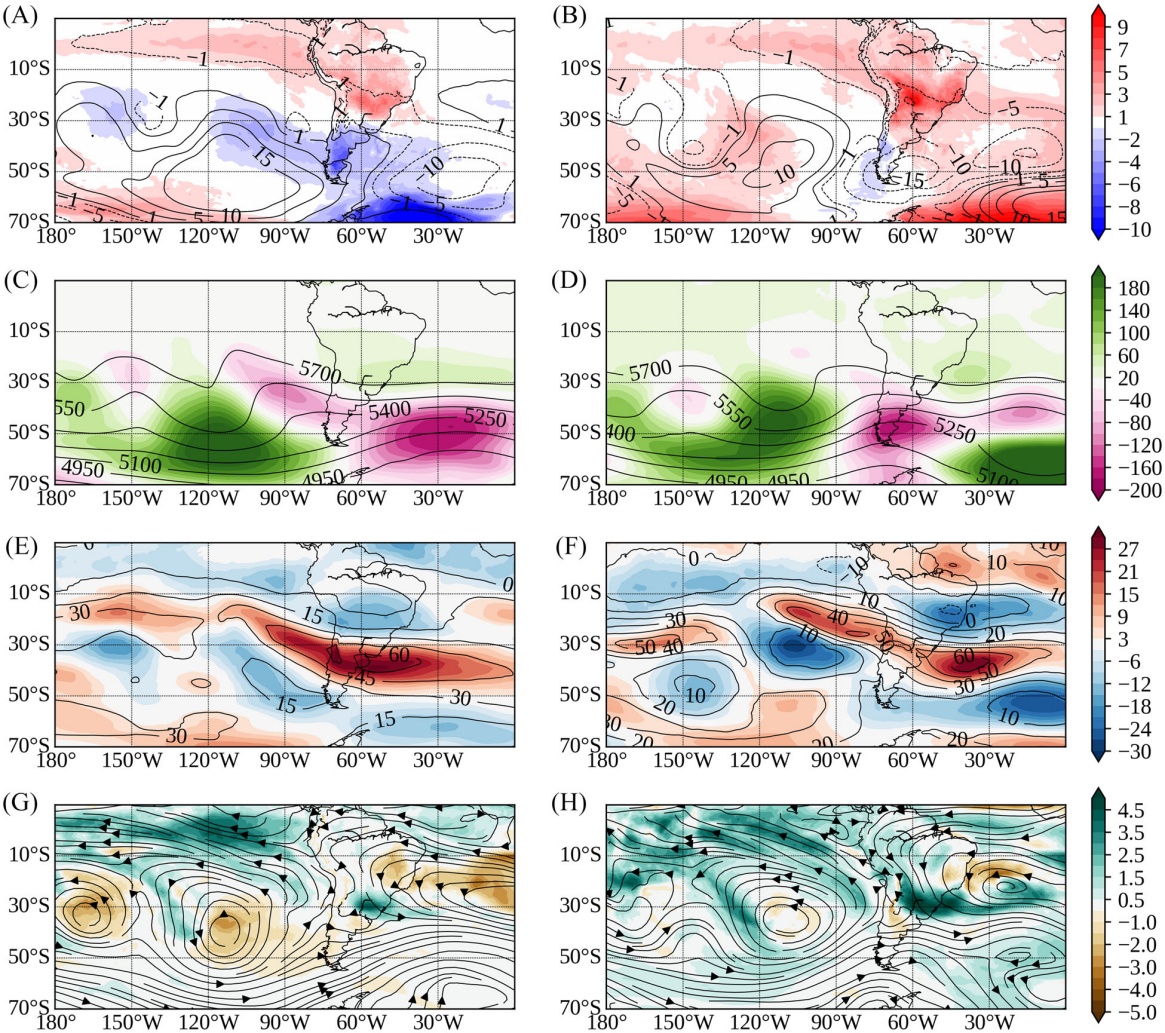
Synoptic conditions for heatwave episodes of (A, C, E, G) September 17–27, 2023 and (B, D, F, H) November 11–18, 2023 for the following variables: (A, B) anomalies of 2 m air temperature (shaded, unit:°C) and sea level pressure (contour, unit: hPa); (C, D) 500‐hPa geopotential height (contour) and 500‐hPa geopotential height anomalies (shaded, unit: mgp); (E, F) 200‐hPa zonal wind speed (contour) and 200‐hPa zonal wind speed anomalies (shaded, unit: m/s); and (G, H) 850‐hPa specific humidity anomaly (shaded, unit: g/kg) and 850‐hPa horizontal wind direction (vector, unit: m/s).

To understand the mechanisms behind the Spring 2023 heatwave events, we analyzed the typical atmospheric circulation patterns. Figure 10 shows the large‐scale features at different pressure levels for the heatwave of September 17–27, 2023, and November 11–18, 2023. Both events exhibited similar large‐scale anomalous patterns. Large positive near‐surface air temperature anomalies dominated central‐north South America, with temperatures exceeding 5°C in Paraguay, northern Argentina, and southern Brazil (Figure 10A,B). The zonal wind circulation at 200 hPa (Figure 10E,F) exhibited a strengthened westerly jet stream extending from the subtropical Pacific Ocean to the mid‐latitude South Atlantic Ocean, with maximum winds over the La Plata basin (exceeding 50 m/s), while weakened westerly winds over central‐north South America were associated with an anticyclonic circulation. At 850 hPa, drier conditions prevailed in central Brazil with negative specific humidity anomalies (Figure 10G,H), whereas southern Brazil exhibited increased humidity driven by northerly winds. A mid‐level ridge associated with positive 500‐hPa geopotential anomalies (Figure 10C,D) enhanced subsidence in central and southeastern Brazil, suppressing precipitation and increasing temperature due to enhanced solar radiation and adiabatic compression.^51^ Both events also featured a Pacific–South American (PSA)‐like wave train that strengthened the upper‐level anticyclonic circulation over southeastern South America, as indicated by the 200‐hPa stream function (Figure S9). This wave train extended from the South Pacific to the South Atlantic Ocean, resembling the positive phase of the first PSA mode^52^ and exhibiting a barotropic‐to‐baroclinic transition as it propagated equatorward.

However, some differences exist between the two heatwaves: the positive near‐surface air temperature anomalies in November were of greater magnitude compared to September; anomalous upper‐level easterly winds were more intense in November than in September, and southern Brazil experienced moister conditions during the November heatwave. These differences highlight variations in intensity and atmospheric dynamics despite the overall similar large‐scale patterns.

## SUMMARY AND CONCLUSIONS

This work provides a detailed analysis of the intense, and some of them extreme, climate events that impacted Brazil throughout the year 2023. That year was marked as the hottest on record in the country until that year, with an annual mean temperature anomaly of +0.84°C relative to the 1991–2020 climatology, with maximum temperature breaking records in many meteorological stations (Araçuaí [MG] registered the highest recorded temperature, reaching 44.8°C). Regionally, the largest positive temperature anomalies were observed in the north, northeast, and southeast regions, while the south showed a more mixed signal, with periods of below and above average temperatures.

In terms of precipitation, the year was characterized by wet and dry extremes. Southern Brazil experienced a substantial increase in precipitation, often associated with cyclones that brought intense rainfall in short time intervals, accounting for a significant portion of monthly rainfall and causing natural disasters. The annual density anomalies indicate that the greater number of cyclogeneses in 2023 in the extreme southwest and northeast of RS was associated with the increased precipitation in South Brazil. This region experienced several daily precipitation extremes, primarily driven by four significant cyclones that occurred in June, July, September, and October. These events caused widespread impacts, with daily rainfall totals often exceeding the 95th climatological percentile and contributing to a great percentage of the accumulated monthly precipitation. The June cyclone, which brought up to 240 mm of rain in eastern RS, caused severe flooding and landslides, while the July event generated strong winds, which reached 24 m/s over the continent, and further disruptions. The September cyclone, the most destructive of the year, resulted in over 3000 people being displaced from their houses. Although the October cyclone was relatively less intense than the other three, it followed similar rainfall patterns, demonstrating the recurring influence of these systems on the region's climate and highlighting the need for effective disaster preparedness.

During the austral summer of 2023, a heavy rainfall event occurred on the northern coast of the state of São Paulo, probably the event with the highest accumulation of rainfall recorded in Brazil in a single day (682.8 mm), which resulted in 65 deaths. This extreme event was associated with different dynamic and thermodynamic factors: The passage of a polar cold front, the action of a mesocyclone in the lower troposphere, a circulation similar to the South American low‐level jet (from the Amazon toward the São Paulo coast), a warmer than normal subtropical South Atlantic Ocean, and the orographic effect of the mountains of the Serra do Mar. Furthermore, the 2023 record exceeded the highest value reported by da Silva et al.^24^, which was 346.6 mm, occurring on January 5, March 10, and December 12 and 31, 1999. Figure 6C from the study by Oda et al.^23^ illustrates that, on days when daily total rainfall exceeded the 99th percentile, there was an anomalous intensification of moisture and warm air transport from the tropics to the central‐north coast of São Paulo. The 850 hPa winds, originating from the western quadrant of the South Atlantic Subtropical Anticyclone, were deflected by the Andes, combining with the anomalous flow from the eastern Equatorial Pacific Ocean toward Southeast Brazil. Additionally, cyclogenesis was observed south of the central‐north coast of São Paulo.

In contrast, northern Brazil, especially the Amazon, experienced a prolonged and intense drought, with significant precipitation deficits in September, October, and November, affecting ecosystems and local communities. The prevailing large‐scale forcings for the austral spring 2023 Amazon drought were the El Niño phenomenon in the equatorial Pacific Ocean, and anomalous SST warming in the tropical North Atlantic Ocean, leading to anomalous subsidence over a large portion of the Amazon due to changes in the zonal (Walker) and meridional (Hadley) circulations.

Heatwaves were a significant phenomenon throughout the year, especially during the austral spring. The WSDI index indicated that some stations in the north and northeast recorded over 100 days of heatwaves, with unprecedented duration and magnitude. Southeastern and central‐western Brazil experienced two intense heatwaves due to a synoptic pattern characterized by an anomalous persistent mid‐tropospheric ridge over central and southeastern Brazil. This system trapped warm air, leading to prolonged high temperatures (positive temperature anomalies up to 4°C) in large areas of southeastern Brazil. The situation might have been related to the El Niño conditions, which typically generates a PSA‐like wave train propagating from the South Pacific to the South Atlantic. The study by Sun, Xue, & Zhou^53^ demonstrated that canonical El Niño events, such as the one observed in 2023, intensify the PSA mode, creating large‐scale circulation anomalies that can influence weather patterns over South America, including the establishment of persistent ridges and heatwaves.

In conclusion, 2023 was a year of extreme climate events in Brazil, reflecting global warming trends and highlighting the need for mitigation and adaptation actions to address climate change and its increasingly severe impacts on the country. These impacts, particularly their social and economic dimensions, represent an important topic for future studies. The growing intensity and frequency of extreme climate events place Brazil in a position of urgency to strengthen response strategies, aiming to reduce vulnerabilities and protect both natural resources and the communities directly affected.

## AUTHOR CONTRIBUTIONS

L.A.P.: Conceptualization; formal analysis; methodology; investigation; visualization; writing—original draft preparation; writing—review, editing; supervision. P.G.B.: Conceptualization; formal analysis; visualization; investigation writing. C.A.S.C., F.C.V.J., H.B.G., and M.L.: Conceptualization; formal analysis; visualization; investigation; writing for Drought in the Amazon region. M.S.C. and C.B.C.: Conceptualization; formal analysis; visualization; investigation; writing for Wet event in the coast of São Paulo state. A.C.N.T., H.R.P., and M.L.S.: Conceptualization; formal analysis; visualization; investigation; writing for the record‐breaking heatwaves of Spring 2023. A.M.P.N., A.A.C., H.A.B., and I.V.G.B.: Conceptualization; formal analysis; visualization; investigation; writing for Extreme precipitation in the South of Brazil. M.S.R., R.P.R., T.A., and G.A.M.S.: Supervision; writing—review and editing.

## COMPETING INTERESTS

The authors declare no conflicts of interest.

## PEER REVIEW

The peer review history for this article is available at https://publons.com/publon/10.1111/nyas.15394.

## Supporting information



Figure S1: Flowchart summarizing the methodology, data used in this study, and the location of the mentioned Brazilian states.
Figure S2: Percentage of missing daily data for conventional stations from INMET (1991−2023) for: (a) compensated average temperature (°C), (b) maximum temperature (°C), and (c) precipitation (mm).
Figure S3: Sea surface temperature anomalies (°C) in the South Atlantic on February 19, 2023, subtracting the reference period of 1991−2020.
Figure S4: 850‐hPa isotachs and magnitude (m/s, shaded) at 00 UTC on February 14−19, 2023.
Figure S5: 250‐hPa streamlines and isotachs (m/s, shaded) at 00 UTC on February 14−17, 2023.
Figure S6: Seasonal anomaly of density of cyclogenesis calculated for 2023 subtracting the reference period of 1991−2020 for (a) JJA and (b) SON. The density unit is the number of cyclones per area (km^2^) × 10^5^ per year.
Figure S7: Monthly mean precipitation (mm/day, blue) from MSWX and soil moisture (m^3^/m^3^, green) from ERA5‐Land over the southern region of Brazil in 2023.
Figure S8: September 1st to November 30th, 2023 daily anomalies of maximum air temperatures for the INMET meteorological stations across the (a) Central‐West and (b) Southeast regions of Brazil period. Anomalies calculated from the 1991−2020 climatology.
Figure S9: Stream function daily anomalies mean at 200 hPa (shaded, unit: 1 × 10^7^ m^2^ s^−1^) and 850 hPa (contour, unit: 1 × 10^7^ m^2^ s^−1^) for heatwave episodes of (a) September 17–27, 2023 and (b) November 11–18.
