# Supplementary material for: Brazil climate highlights 2023
Source: Ann N Y Acad Sci. 2025 Jun 15;1549(1):120–38. doi: 10.1111/nyas.15394 (PMC12309436; doi:10.1111/nyas.15394)
Supplement: Supplementary file 1 — Figure S1: Flowchart summarizing the methodology, data used in this study, and the location of the mentioned Brazilian states. Figure S2: Percentage of missing daily data for conventional stations from INMET (1991−2023) for: (a) compensated average temperature (°C), (b) maximum temperature (°C), and (c) precipitation (mm). Figure S3: Sea surface temperature anomalies (°C) in the South Atlantic on February 19, 2023, subtracting the reference period of 1991−2020. Figure S4: 850‐hPa isotachs and magnitude (m/s, shaded) at 00 UTC on February 14−19, 2023. Figure S5: 250‐hPa streamlines and isotachs (m/s, shaded) at 00 UTC on February 14−17, 2023. Figure S6: Seasonal anomaly of density of cyclogenesis calculated for 2023 subtracting the reference period of 1991−2020 for (a) JJA and (b) SON. The density unit is the number of cyclones per area (km2) × 105 per year. Figure S7: Monthly mean precipitation (mm/day, blue) from MSWX and soil moisture (m3/m3, green) from ERA5‐Land over the southern region of Brazil in 2023. Figure S8: September 1st to November 30th, 2023 daily anomalies of maximum air temperatures for the INMET meteorological stations across the (a) Central‐West and (b) Southeast regions of Brazil period. Anomalies calculated from the 1991−2020 climatology. Figure S9: Stream function daily anomalies mean at 200 hPa (shaded, unit: 1 × 10^7^ m2 s−1) and 850 hPa (contour, unit: 1 × 10^7^ m2 s−1) for heatwave episodes of (a) September 17–27, 2023 and (b) November 11–18. [file NYAS-1549-120-s001.docx]

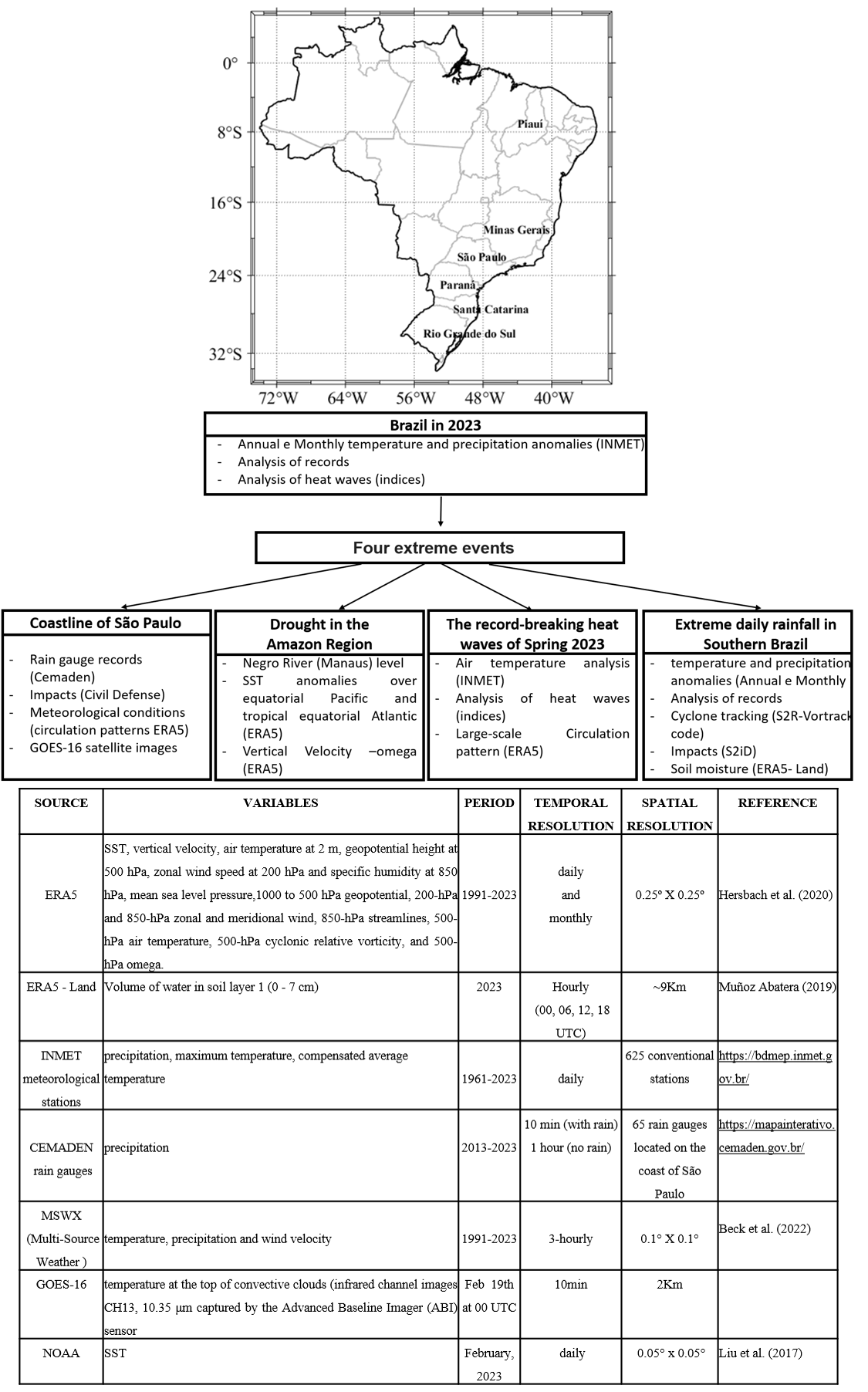
**Figure S1:** Flowchart summarizing the methodology, data used in this study and the location of the mentioned Brazilian states.


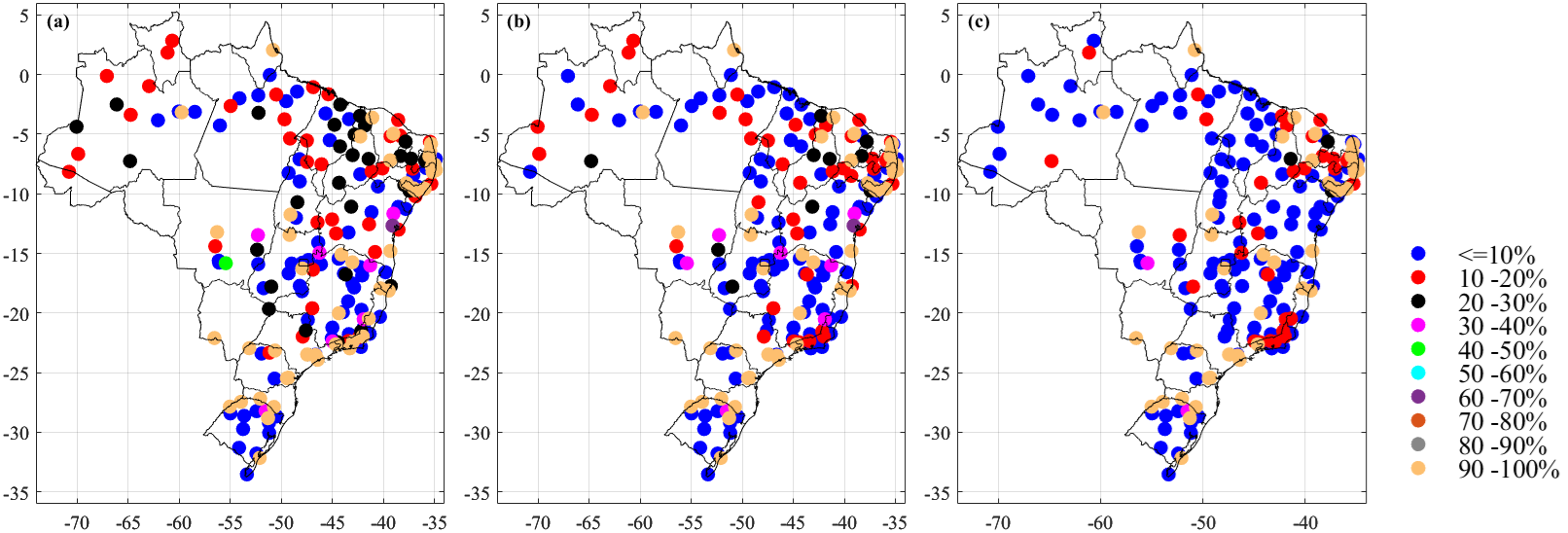
**Figure S2:** Percentage of missing daily data for conventional stations from INMET (1991-2023) for: (a) compensated average temperature (^o^C) (b) maximum temperature (^o^C) and (c) precipitation (mm).


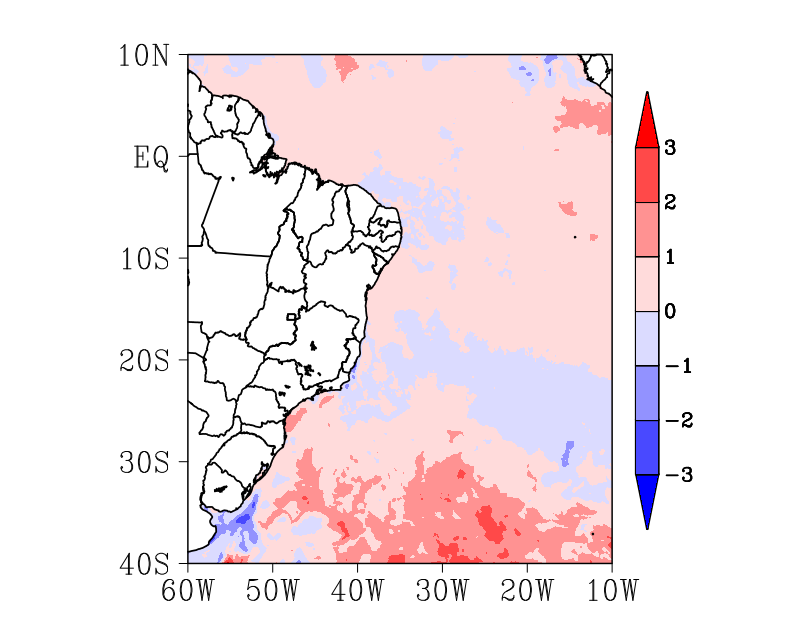


**Figure S3:** Sea surface temperature anomalies (°C) in the South Atlantic on February 19, 2023, subtracting the reference period of 1991-2020.


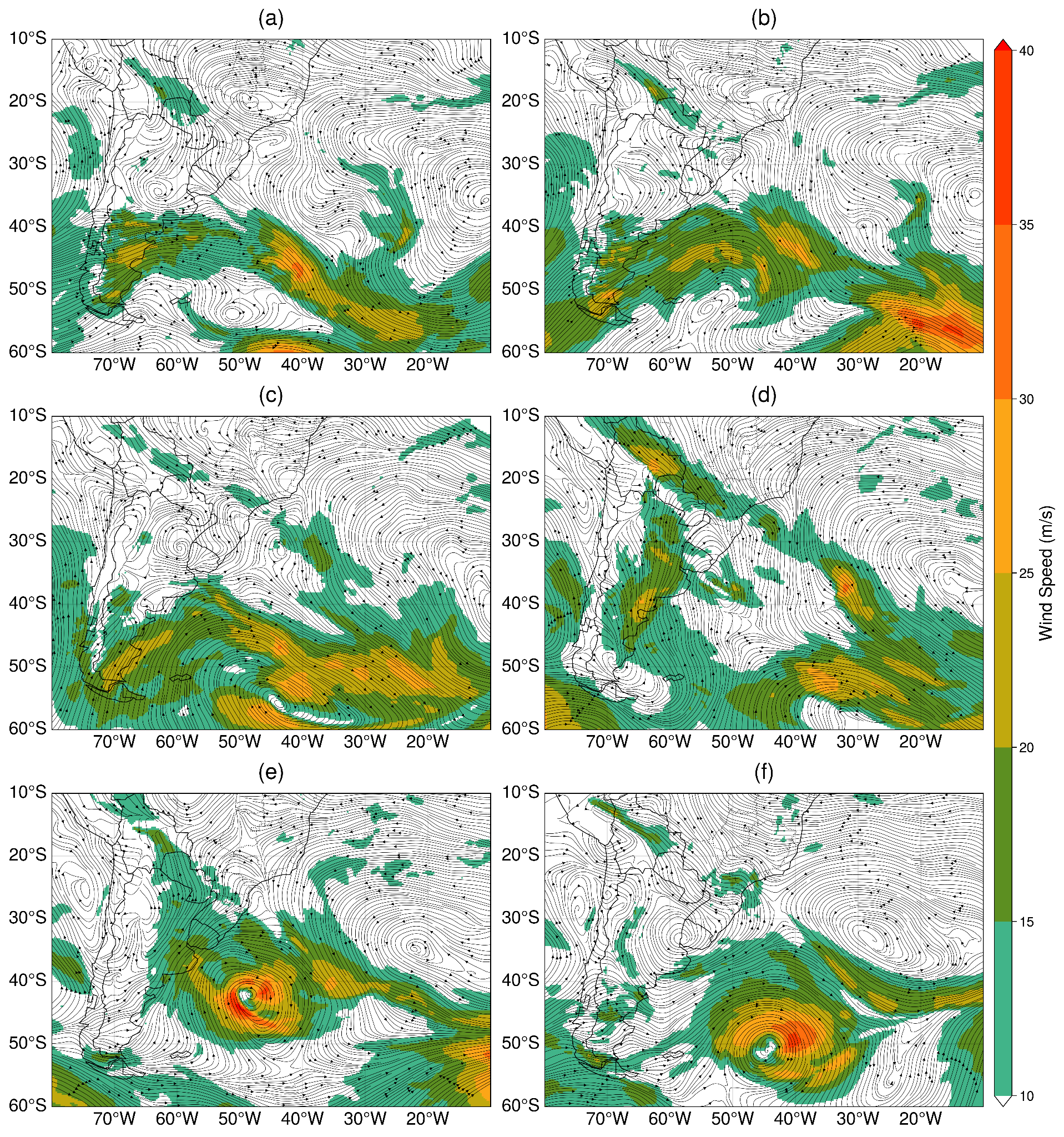


**Figure S4:** 850-hPa isotachs and magnitude (m/s, shaded) at 00 UTC on February 14 to 19, 2023.


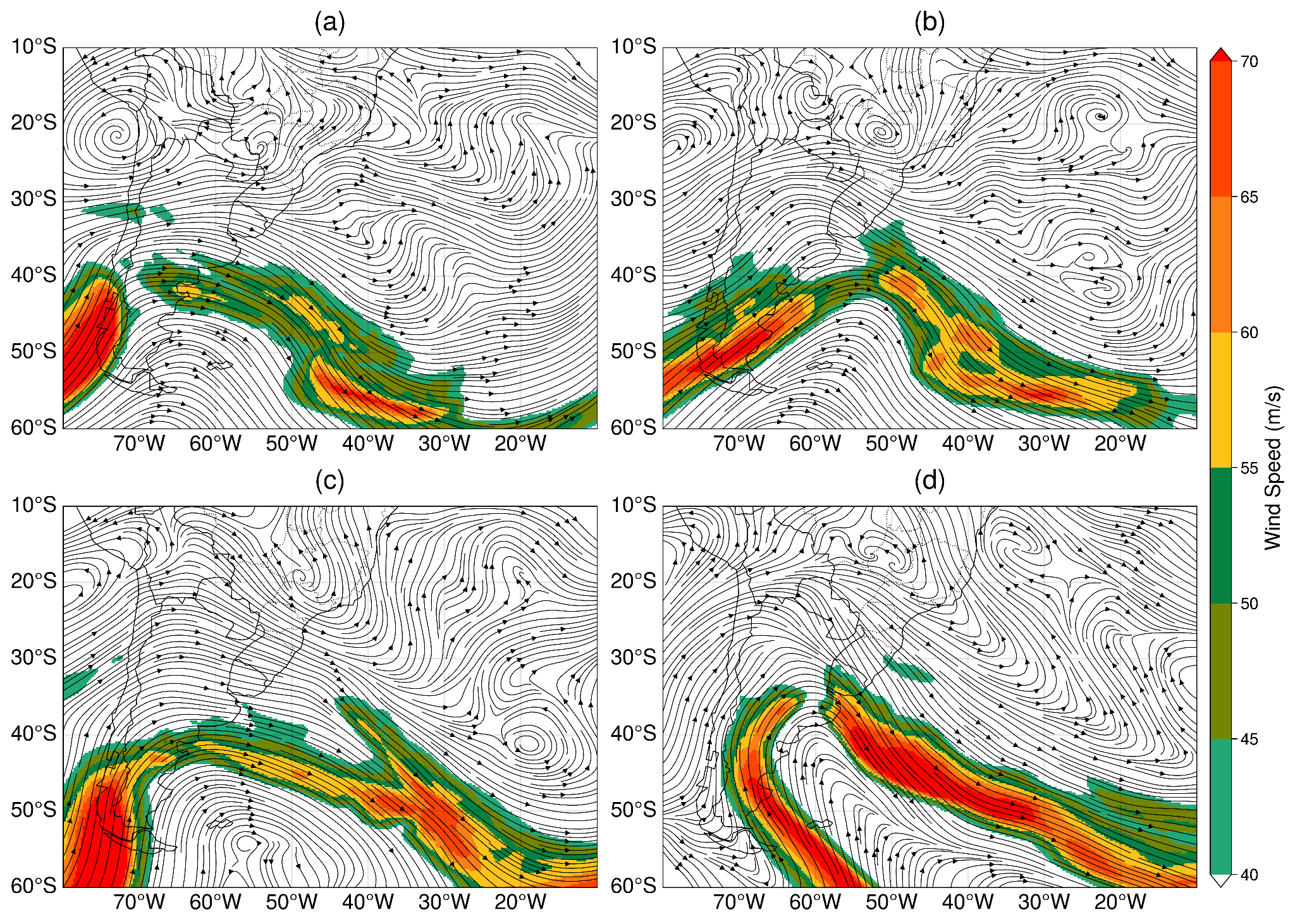


**Figure S5:** 250-hPa streamlines and isotachs (m/s, shaded) at 00 UTC on February 14 to 17, 2023.


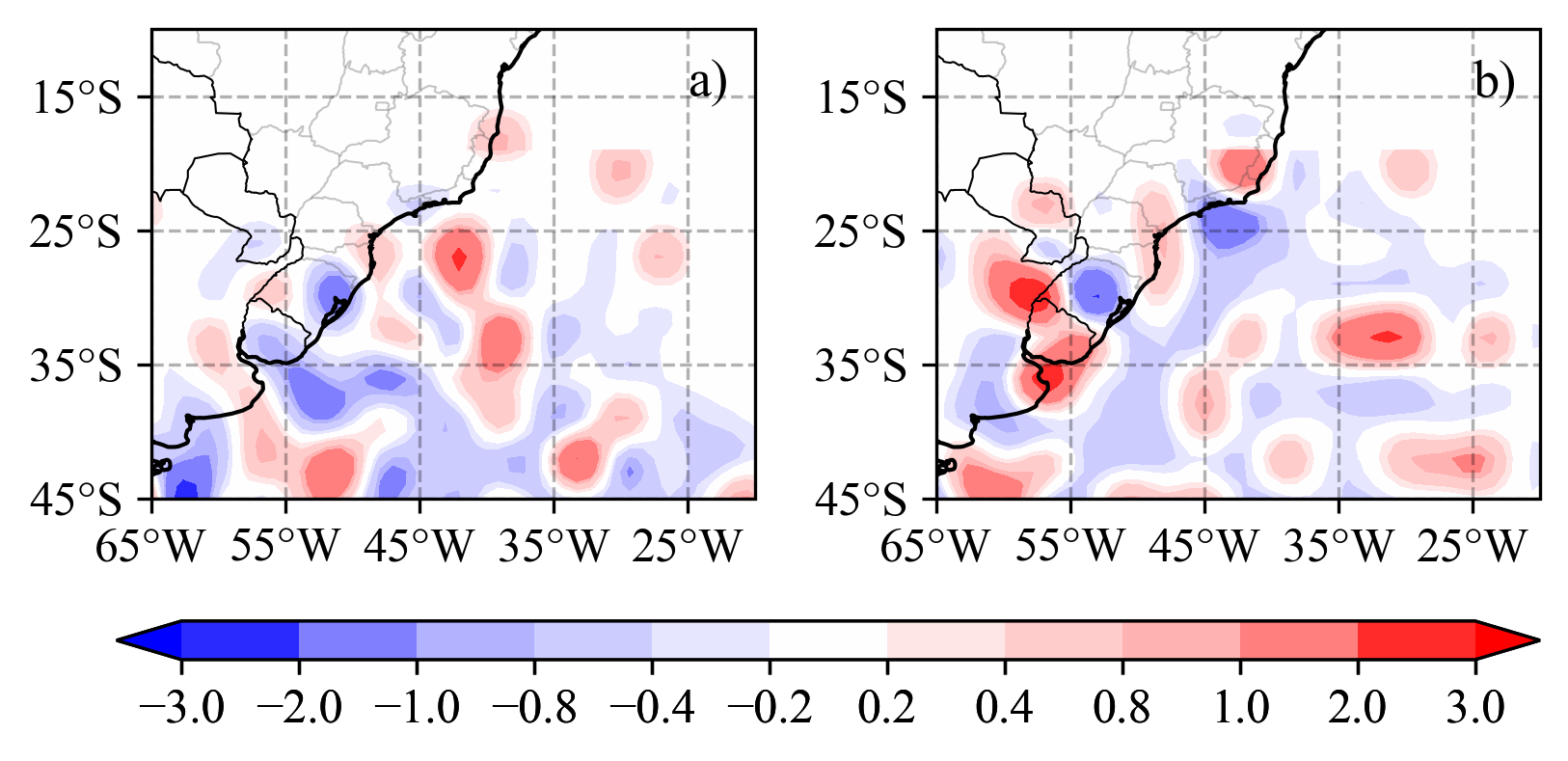


**Figure S6:** Seasonal anomaly of density of cyclogenesis calculated for 2023 subtracting the reference period of 1991-2020 for a) JJA and b) SON. The density unit is the number of cyclones per area (km^2^) × 10^5^ per year.


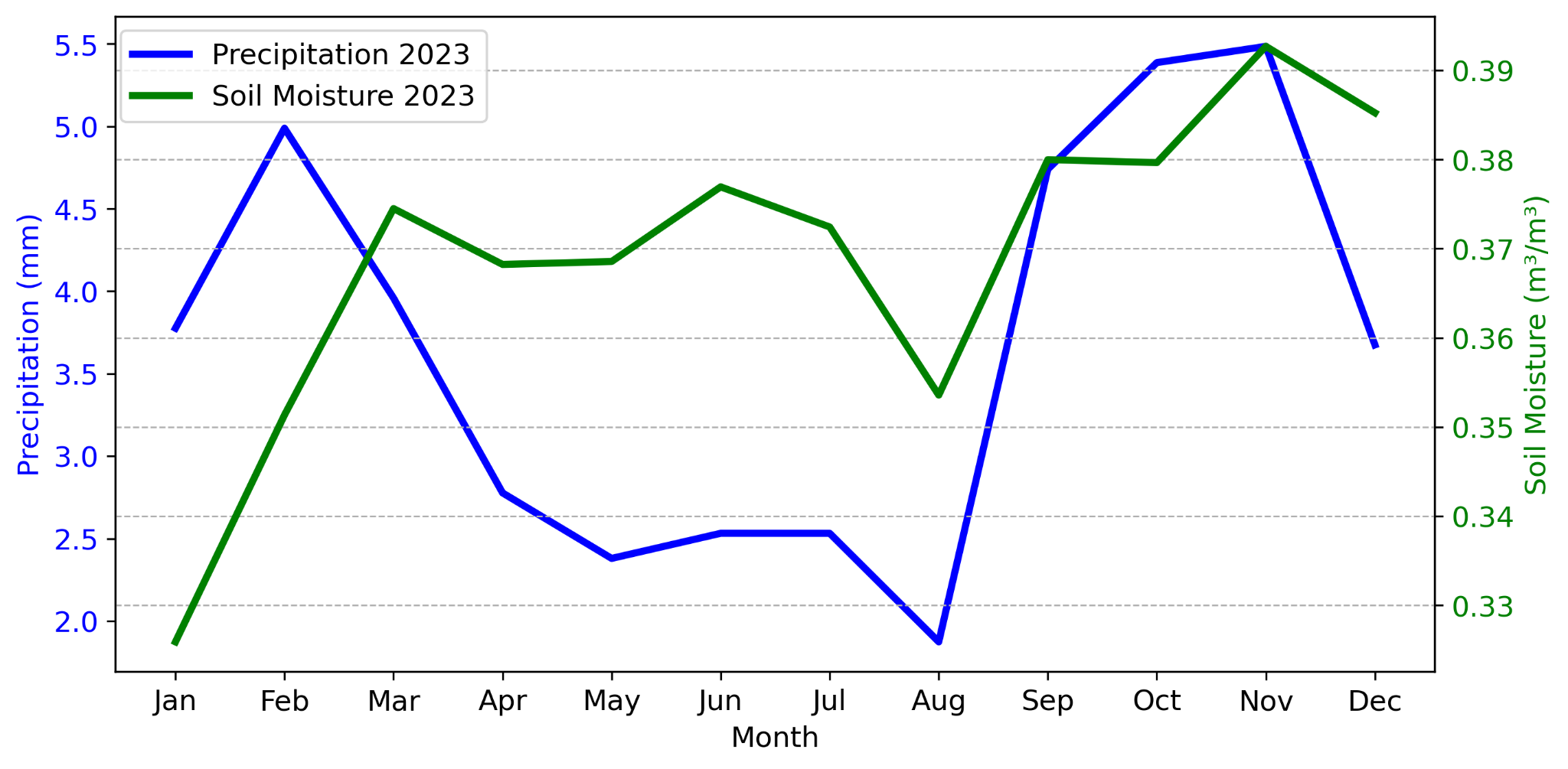


**Figure S7:** Monthly mean precipitation (mm/day, blue) from MSWX and soil moisture (m³/m³, green) from ERA5-Land over the southern region of Brazil in 2023.


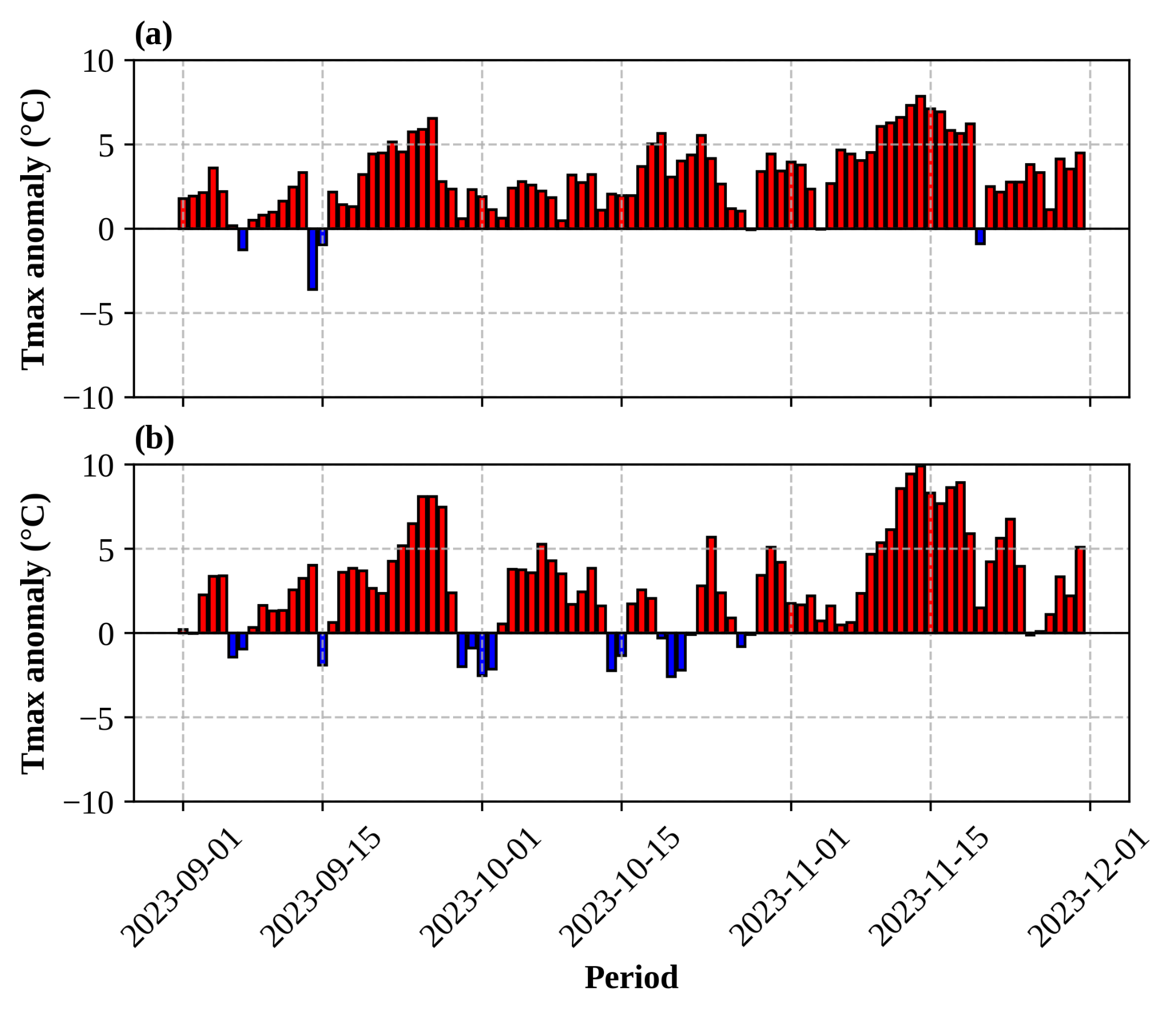


**Figure S8:** September 1^st^ to November 30^th^, 2023 daily anomalies of maximum air temperatures for the INMET meteorological stations across the **a)** Central-West and **b)** Southeast regions of Brazil period from. Anomalies calculated from the 1991-2020 climatology.

**
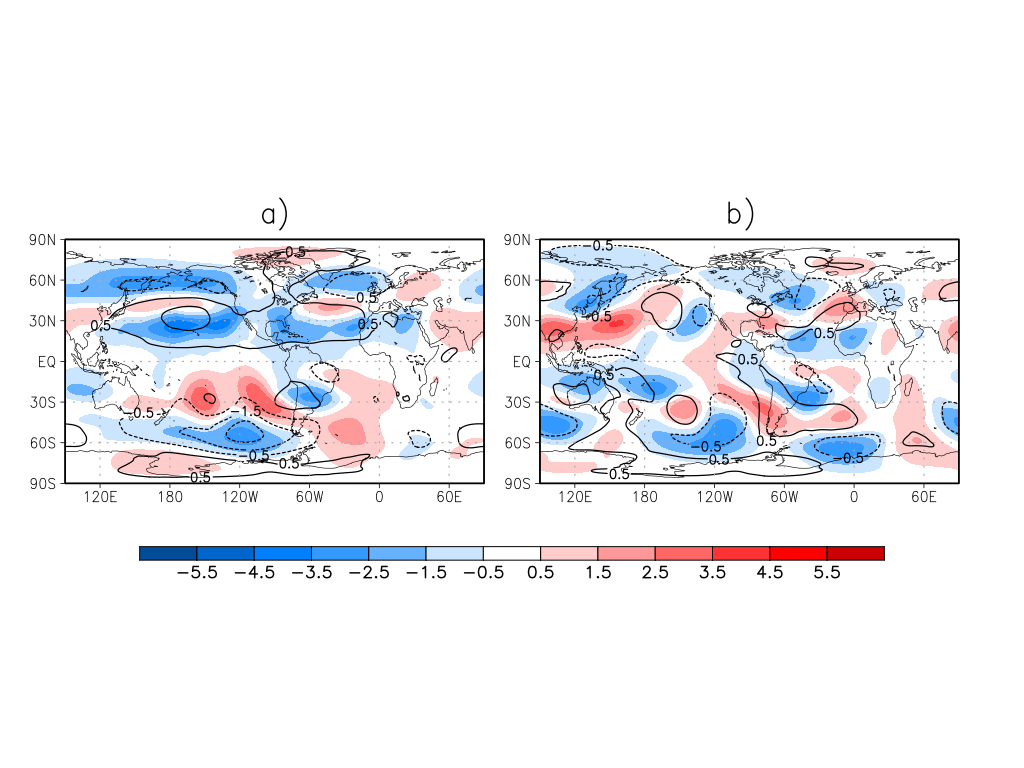
**

**Figure S9**: Stream function daily anomalies mean at 200 hPa (shaded, unit: 1 x 10^7^ m^2^ s^-1^) and 850 hPa (contour, unit: 1 x 10^7^ m^2^ s^-1^) for heatwave episodes of (a) September 17–27, 2023 and (b) November 11–18.
